# Dual transcriptome of the immediate neutrophil and *Candida albicans* interplay

**DOI:** 10.1186/s12864-017-4097-4

**Published:** 2017-09-06

**Authors:** Maria J. Niemiec, Christian Grumaz, David Ermert, Christiane Desel, Madhu Shankar, José Pedro Lopes, Ian G. Mills, Philip Stevens, Kai Sohn, Constantin F. Urban

**Affiliations:** 1 Department of Clinical Microbiology, Umeå Centre for Microbial Research (UCMR) & Laboratory of Molecular Infection Medicine Sweden (MIMS), Umeå University, Umea, Sweden; 2 Fraunhofer Institute for Interfacial Engineering and Biotechnology IGB, Stuttgart, Germany; 3 Institute of Clinical Microbiology, Immunology and Hygiene, University Hospital Erlangen, Friedrich-Alexander-Universität Erlangen-Nürnberg, Erlangen, Germany; 4 Prostate Cancer Research Group, Center of Molecular Medicine Norway (NCMM), Oslo, Norway; 5 Department of Molecular Oncology, Institute of Cancer Research, Radium Hospital, Oslo, Norway; 6 PCUK/Movember Centre of Excellence for Prostate Cancer Research, Centre for Cancer Research and Cell Biology (CCRCB), Queen’s University Belfast, Belfast, UK; 7 University of Stuttgart IGVP, Stuttgart, Germany; 8 Center for Integrative Bioinformatics Vienna, Max F. Perutz Laboratories, University of Vienna, Vienna, Austria; 9 Present Address: Leibniz Institute for Natural Product Research and Infection Biology - Hans Knöll Institute (HKI), Jena, Germany & Center for Sepsis Control and Care (CSCC), Jena, Germany; 10 Present Address: Division of Medical Protein Chemistry, Department of Translational Medicine, Lund University, Malmö, Sweden; 11 Present Address: The Weatherall Institute of Molecular Medicine, University of Oxford, Oxford, UK

**Keywords:** *Candida*, Neutrophils, Dual transcriptome, Extracellular traps, NOD-like receptor pathway, Nutritional immunity, Morphotype

## Abstract

**Background:**

Neutrophils are traditionally considered transcriptionally inactive. Compared to other immune cells, little is known about their transcriptional profile during interaction with pathogens.

**Methods:**

We analyzed the meta-transcriptome of the neutrophil-*Candida albicans* interplay and the transcriptome of *C. albicans* challenged with neutrophil extracellular traps (NETs) by RNA-Seq, considering yeast and hypha individually in each approach.

**Results:**

The neutrophil response to *C. albicans* yeast and hyphae was dominated by a morphotype-independent core response. However, 11 % of all differentially expressed genes were regulated in a specific manner when neutrophils encountered the hyphal form of *C. albicans*. While involving genes for transcriptional regulators, receptors, and cytokines, the neutrophil core response lacked typical antimicrobial effectors genes. Genes of the NOD-like receptor pathway, including *NLRP3*, were enriched.

Neutrophil- and NET-provoked responses in *C. albicans* differed. At the same time, the *Candida* transcriptome upon neutrophil encounter and upon NET challenge included genes from various metabolic processes and indicate a mutual role of the regulators Tup1p, Efg1p, Hap43p, and Cap1p. Upon challenge with neutrophils and NETs, the overall *Candida* response was partially morphotype-specific. Yet again, actual oppositional regulation in yeasts and hyphae was only detected for the arginine metabolism in neutrophil-infecting *C. albicans*.

**Conclusions:**

Taken together, our study provides a comprehensive and quantitative transcript profile of the neutrophil–*C. albicans* interaction. By considering the two major appearances of both, neutrophils and *C. albicans*, our study reveals yet undescribed insights into this medically relevant encounter. Hence, our findings will facilitate future research and potentially inspire novel therapy developments.

**Electronic supplementary material:**

The online version of this article (10.1186/s12864-017-4097-4) contains supplementary material, which is available to authorized users.

## Background

Neutrophils are essential to fight off microbial infections using a well-orchestrated set of intra- and extracellular antimicrobial processes [1]. These phagocytes mature in the bone marrow and are constantly released into the blood stream. In circulation, they are fully matured. Their lifespan is comparably short between one and several days [2, 3]. At the site of infection, pathogen-associated molecular patterns (PAMPs) are at first recognized by pattern recognition receptors (PRRs) on the surface of neutrophils. Of all fungi-sensing PRRs, approximately 50% are present on neutrophils such as Toll-like receptors, C-type lectin receptors, complement receptor CR3, and Fc receptors [4–6]. Most of these PRRs recognize fungal cell wall components, like e.g. mannan and β-1,3-glucan [7]. The next step of the neutrophil-microbe interaction is phagocytosis. Phagosomes fuse with cytoplasmic vesicles (granules) to form a highly hostile environment that contains antimicrobial enzymes and peptides as well as reactive oxygen species (ROS) [8]. Within mature phagosomes neutrophils eradicate pathogens efficiently [1, 9, 10]. Neutrophil granule content is in addition released into the extracellular space to destroy not internalized microbes [11]. Neutrophil extracellular traps (NETs) are a second external killing mechanism [12]. Unlike during degranulation, neutrophils die during NET formation [13]. Before that, a controlled loss of intracellular membrane integrity allows the contents of formerly separated compartments to mix. Therefore NET fibers contain nuclear chromatin decorated with granular and cytoplasmic effectors [14–16]. In addition to directly affecting the microbial invader, neutrophils are able to release cytokines in order to recruit or manipulate other immune cells [17, 18].

A pathogen frequently encountered by neutrophils is *Candida albicans*. Today, *Candida ssp*. cause most of the opportunistic fungal infections worldwide. Nevertheless, *C. albicans* is a commensal in more than 50% of the human population [19]. It colonizes different mucosal niches, such as for instance the gastrointestinal (GI) tract, without causing apparent symptoms. However, upon perturbance of immune barriers or functions, *C. albicans* can turn into a *bona fide *pathogen [20]. The fungus causes a wide range of diseases from superficial, invasive to systemic infections. Invasive forms of candidiasis are often correlated to extremes of age, organ transplantation, blood cancer and HIV/AIDS. Epidemiological studies show that *Candida* infections obtained in intensive care units can be fatal in up to 50% of the cases [21, 22].

*C. albicans* pathogenicity is based on a variety of features, amongst which the ability to change morphotypes is essential. *C. albicans* can grow as ovoid-shaped, budding yeast or as filamentous hyphae. A considerable number of studies have investigated the role of the respective morphotypes during in vivo infections. It was shown that mutants locked in one or the other morphotype are avirulent [23]. Furthermore, it was revealed that both morphotypes can be isolated from infection sites [24]. Human neutrophils are able to eradicate both morphotypes by intra- and extracellular mechanisms [15, 25]. Several studies have been performed to elucidate the antifungal mode of action of NETs. The protein complex calprotectin, a heterodimer composed of the proteins S100A8 and S100A9, is a zinc and manganese chelator and was found to be associated with NETs after NETosis. Among the NET-associated host effectors, calprotectin is the key effector in NETs towards fungal pathogens [16, 26–29].

It is likely due to the short life span and completed maturation of neutrophils that their transcriptional capacity is underestimated resulting in a small number of transcriptional studies in neutrophils [30]. Early work revealed RNA and protein synthesis in neutrophils in the 1970ies [31]. In 2004, a microarray-based analysis of the neutrophil transcriptional response was performed using live, opsonized *Escherichia coli*, lipopolysaccharide (LPS), and formyl-Met-Leu-Phe (fMLF) as stimuli [32]. Transcriptional responses of neutrophils and monocytes towards *C. albicans* were analyzed by microarray confirming a transcriptional activation of neutrophils by the fungus [33]. Remarkably, the response involved genes acting in cell communication, e.g. cytokines, but lacked typical antimicrobial effectors needed for a prompt deactivation of pathogens. More recently, a study compared the transcriptional effects of priming agents TNF-α and GM-CSF on human neutrophils using RNA-Seq [34]. Many studies have analyzed the gene expression in *C. albicans* reacting to a variety of stimuli. Biotic stimuli were amongst others full blood [35], granulocytes isolated from blood [36] and macrophages [37, 38].

The present study analyzed the transcriptional response of human neutrophils and *C. albicans* during their interaction in greater detail by employing RNA-Seq. First, we performed in vitro infections of neutrophils by *C. albicans* yeast or *C. albicans* hyphae and investigated the early transcriptional response from both interaction partners. *Candida* yeast and hyphae are both susceptible towards NETs [15]. However, it is incompletely understood how *C. albicans* transcriptionally responds to NET-mediated stress. Thus, we challenged *C. albicans* yeasts and hyphae with NETs and analyzed their transcriptional response.

Among the investigated time points we found the strongest transcriptional response of neutrophils to *C. albicans* after 60 min. Genes induced in neutrophils code for proteins involved in transcriptional regulation, gene expression, cytokine production, stress responses, and apoptosis delay. The majority of 318 differentially expressed genes (DEGs) was up-regulated. Notably, we found approximately 11% of the DEGs to be morphotype-specifically regulated upon encounter of *C. albicans* hyphae. In contrast, the transcriptional response of *C. albicans* was spread more evenly over the entire time course analyzed. Overall, *C. albicans*’ response towards neutrophils was dominated by metabolic genes which are controlled by the transcriptional regulators Tup1p, Efg1p, Hap43p, and Cap1p. No apparent morphotype specificity was discovered in *C. albicans* challenged with NETs and overall the transcriptional response of *C. albicans* to NETs was very different from the response to intact neutrophils. Our findings give detailed insight into the neutrophil–*C. albicans* interaction and contribute to the understanding of this exceptional host-pathogen interaction.

## Results

### Transcriptional response of neutrophils to *C. albicans* occurs predominantly after 60 min and is dominated by a morphotype-independent core response

**Fig. 1:**
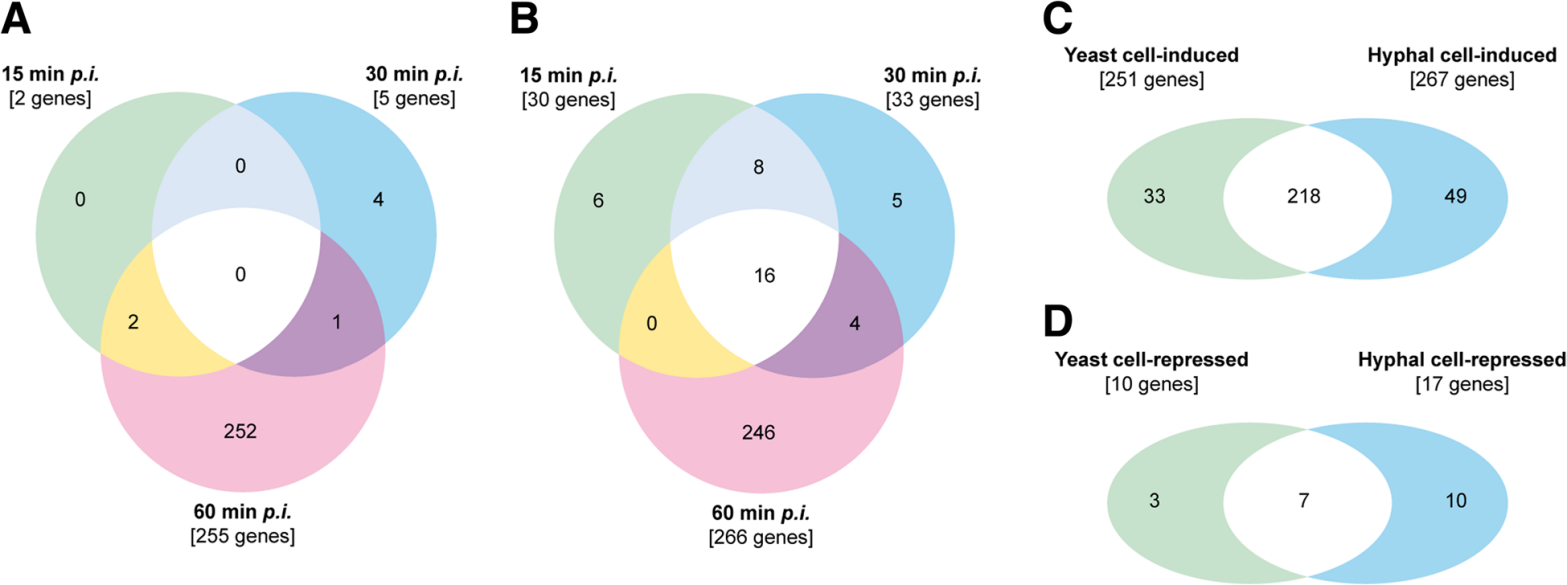
Overlaps of DEGs in neutrophils infected with *C. albicans* yeast and hyphae. Overlap of DEGs in neutrophils infected with *C. albicans* yeast (**a**) and hyphae (**b**) over time. Overlap of morphotype-specific neutrophil response of induced (**c**) and repressed (**d**) DEGs. Samples from two independent experiments using different blood donors were analyzed, *n* = 2

Since *C. albicans* is a polymorphic pathogen and mechanisms applied by neutrophils to discriminate between the *C. albicans* morphotypes remain incompletely understood [39–41], we analyzed transcriptional changes in neutrophils isolated from healthy donors upon yeast and hypha infection separately. DEGs were determined through comparison of infected and uninfected neutrophils. We have shown earlier that quantification based on dry mass was preferable to determine comparable amounts of *C. albicans* yeasts and hyphae [39]. In the present study we infected with similar dry masses, which correlated well with surface area of yeast and hyphal cells resulting in comparable infectious loads and contact sites for neutrophils. The infection of neutrophils was analyzed after 15, 30 and 60 min to represent different stages of in vitro infection: recognition and adhesion, early phagocytosis, and entire phagocytosis of yeast or wrapping of hyphae, respectively. For both infections, neutrophil DEGs were most abundant at 60 min *post infection (p.i.)* indicating that the immediate attack of neutrophils does not majorly require de novo synthesis of effector molecules (Fig. 1a and b, yeasts and hyphae, respectively). Of note, the purity of our neutrophil preparations was verified by flow cytometry (Additional file 1: Figure S1). CD11b-positive and MHC class II-negative cells were considered as neutrophilic granulocytes. Their share was consistently higher than 93%. CD3-positive T cells and CD19-positive B cells accounted for less than 1% of the purified cells. The majority of cells carried-over were eosinophilic granulocytes, showing characteristic high side scatter and auto-fluorescence properties, whereas CD14-positive monocytes constituted below 0.1% of the cell suspension.

For the neutrophil response, an average of 35.4 million reads was sequenced. Of those, 25.9 million reads were uniquely mapped (73%) and therefore could be utilized for subsequent gene expression analyses Additional file 2: Table ST1).

As an overall trend, the transcriptional response of neutrophils peaked after 60 min, independent of the *Candida* morphotype used for the infection (Fig. 1a and b, Additional file 3: Figure S2A, Additional file 4: TableST2). In detail, slight differences could be detected: Nearly all DEGs in yeast-infected neutrophils were exclusively affected at 60 min *p.i.* and no DEGs were found to be constitutively affected throughout the entire infection (Fig. 1a). In contrast, a group of 16 DEGs, all up-regulated, appeared at all times in the hypha-infected neutrophils (Fig. 1b).

When comparing all the neutrophil genes up- and down-regulated in response to either yeasts or hyphae, the majority belonged to a 225 gene-containing morphotype-independent ‘core neutrophil response’. Of those, 218 were up-regulated and only 7 down-regulated (Fig. 1c and d). When grouping the 225 genes in 9 major classes according to function, the relative abundance indicated a high level of cellular reprogramming, induction of gene expression, cytokine production and stress response (Table 1).

**Table 1:** Transcriptional re-shaping of the neutrophil during *C. albicans* infection

Cellular function	%
Transcription factors and regulators	15.6
Gene expression	11.5
Receptors	6.4
Signaling	22.0
Cytokines	6.4
Adhesion and migration	2.8
Membrane trafficking & phagocytosis	6.4
Metabolism, stress response, transport	17.4
Uncharacterized	11.5

Entity of neutrophil DEGs affected during infection with *C. albicans* yeast and hyphae were assigned to 9 different cellular functions and the relative abundance of DEGs in this group was determined. Assignment to function was done using NCBI, KEGG and GeneCards® databases

In addition to function, the union set of all neutrophil DEGs, which comprise 318 genes, were also clustered by expression pattern (Additional file 5: Figure S3). Eight different clusters were identified by QT-Clustering using Mayday and most DEGs were assigned to cluster 8 containing genes with increasing expression from 15 min to 60 min (Additional file 5: Figure S3: cluster 8) [42]. In three clusters, we identified 36 of the DEGs to be morphotype-specifically regulated in hypha-infected neutrophils (Additional file 5: Figure S3: cluster 1, 3 + 7). Those genes code for cytokines, receptors, potential PRRs, and transcription regulators. As these are proteins of versatile function and a noticeable proportion of these specifically regulated genes has not been assigned to a particular function in neutrophils yet, their abundance does not point towards one clear key mode of action of neutrophils battling *C. albicans* hyphae.

In summary, both quantity and intensity of the transcriptional response of human neutrophils towards *C. albicans* yeasts and hyphae increased from 15 min to 60 min *p.i.*. This indicates that different stages of interplay might be implied. The immediate early response of neutrophils to *Candida* involves minor but distinct transcriptional activity, but might be executed mostly by posttranslational events. In contrast, later responses seem to require de novo synthesis of proteins. A concise but noticeable proportion of the neutrophil DEGs was regulated in a morphotype-specific manner upon encounter of *C. albicans* hyphae.

### Expression of transcription regulators, PRRs and cytokine receptors promotes inflammatory responses and a switch from migration to battle

**Fig. 2:**
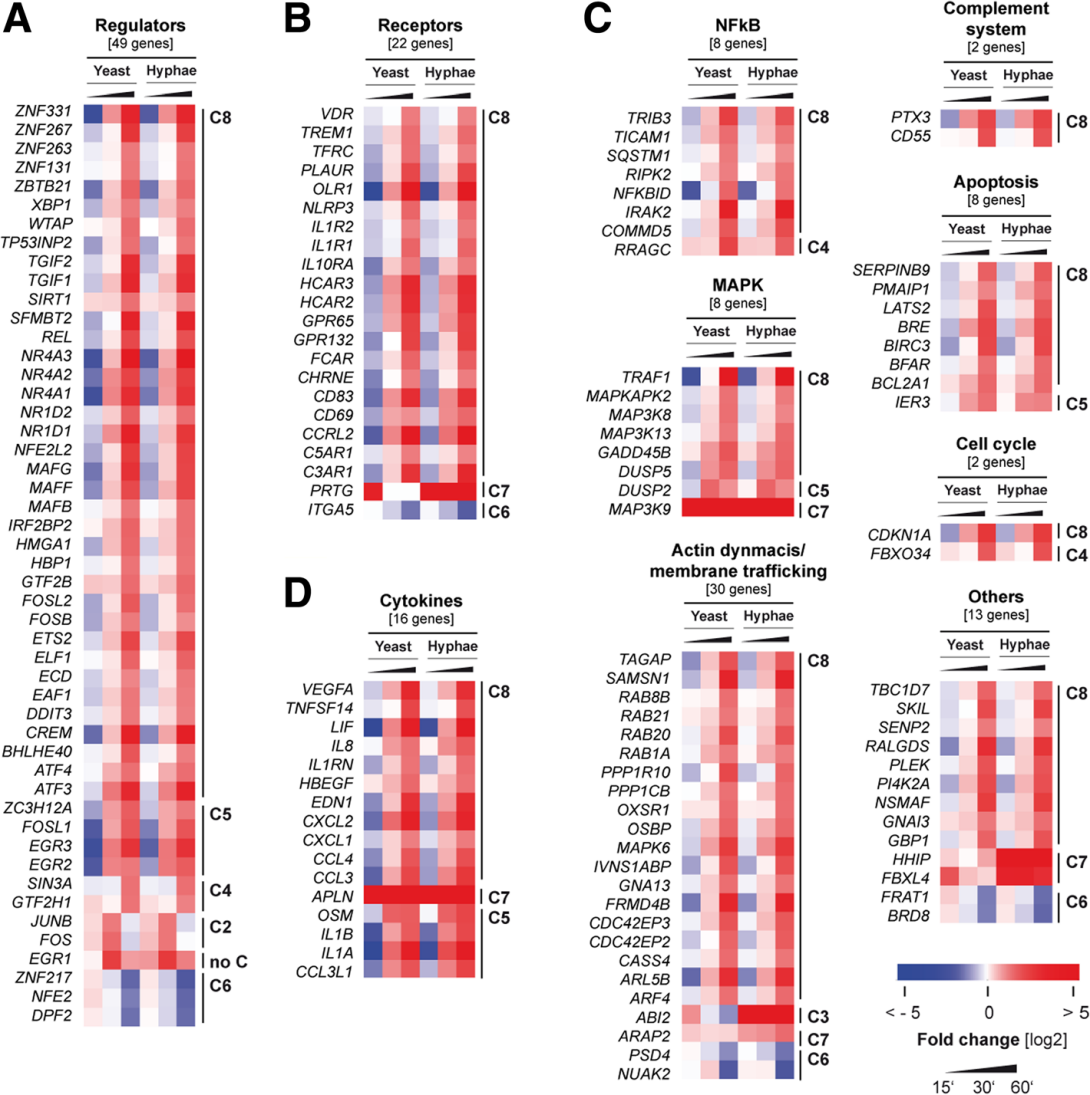
Regulation of regulator, receptor, cytokine, and signaling DEGs affected during neutrophil-*C. albicans* encounter. Neutrophil DEGs from the ‘neutrophil core response’ upon *C. albicans* infection were grouped according to the function of their coded protein. Those coding for transcriptional regulators (**a**), receptors (**b**), signaling effectors (**c**), and cytokines (**d**) are plotted. The transcript profile is displayed for 15 min, 30 min and 60 min *p.i.* with yeast or hyphae. Red indicates up-regulation, blue indicates down-regulation. Samples from two independent experiments using different blood donors were analyzed, *n* = 2

The group of genes involved in transcriptional regulation contained 49 genes (Fig. 2a) clustered by their dynamic expression patterns (Additional file 5: Figure S3). The majority of differentially expressed regulator genes belonged to cluster 8 with increasing transcript levels over time. Many of these regulator genes were zinc-finger repressors or leucine zipper motif-containing factors. These regulators have been described to play a role during inflammation and oxidative stress responses. Remarkably, genes of all members of the FOS family (*FOS*, *FOSB*, *FOSL1*, *FOSL2*) were differentially expressed. The coded proteins of *FOSL2* and *FOSB* dimerize with JUN to form the transcription factor complex AP-1 [43]. Interestingly, *EGR1*, even though considered as unclustered, showed highest transcript levels after 30 min of infection, therefore being the most induced DEG at this time point (Additional file 6: Table ST3). A role for transcriptional activator EGR1 during inflammatory responses of neutrophils has been described [44].

Further, we revealed several transcription regulator genes to be expressed at first and repressed at later time points, such as *DPF2* (Additional file 5: Figure S3, cluster 6). The coded protein is crucial for induction of apoptosis upon e.g. starvation. Interestingly, we identified a higher relative abundance of transcription regulator genes among the most affected neutrophil genes during yeast infection compared to hypha infection (Additional file 6: Table ST3).

The group of receptor genes we found to be differentially expressed in neutrophils consists of 22 genes (Fig. 2b). The majority of these genes was down-regulated at first but subsequently induced to statistically significant levels at 60 min *p.i.* (Additional file 5: Figure S3, cluster 8). For instance, *IL1R1* codes for the IL-1 receptor type I and is pro-inflammatory. *C3AR1* and *C5AR1* are the genes of the complement component 3a receptor 1 and complement component 5a receptor [45]. Interestingly, we identified the gene *TREM1* and *NLRP3* to be up-regulated. TREM-1 is a pro-inflammatory receptor that interacts with the TLR4-mediated response [46]. *NLRP3* encodes for Nalp3 (or NLRP3); an intracellular PRR capable of triggering inflammasome activation [47].

In summary, the group of differentially expressed transcriptional regulators indicates induction of an inflammatory and oxidative stress response and simultaneous repression of apoptosis, whereas the regulation pattern of neutrophil receptor genes indicates that neutrophils prepare to phase out migration and engage in direct interaction with the fungal pathogen.

### Major immunological pathways and cytoskeleton are affected in *Candida*-infected neutrophils

The biggest group of DEGs in *Candida*-infected neutrophils were genes whose products function in cell signaling. The group consisted of 71 genes (Fig. 2c) and a number of signaling pathways were affected: NfκB, MAPK, JAK-STAT, actin dynamics and membrane trafficking, complement system, cell cycle, and apoptosis were most evident.

We identified 8 DEGs related to the NfκB pathway and the same number of genes involved in the MAPK pathway. The majority of these 16 genes showed increased transcript levels at 60 min *p.i.* (Additional file 5: Figure S3, cluster 8). NfκB-related genes were for instance *IRAK2* coding for IL-1 receptor-associated kinase 2 and *TICAM1* coding for the toll-like receptor molecule adaptor 1 used by TLR3 (not present on neutrophils) and TLR4. DEGs involved in the MAPK pathway were for example *MAP3K8* and *MAPKAPK2.* The products of both genes are mediators in TLR signaling.

A group of 30 DEGs codes for signaling proteins involved in actin dynamics and membrane trafficking most probably representing cytoskeleton rearrangements due to direct interaction with fungal cells (Fig. 2c). Among those we found 6 small GTPase genes associated to vesicular trafficking, as well as genes coding for proteins of the Ras superfamily of GTPases and Rho GTPase activating or binding proteins that play a role in F-actin and pseudopodia formation.

In addition, we found 7 apoptosis-related genes clustered together that were exclusively statistically significantly up-regulated at 60 min *p.i.* (Additional file 5: Figure S3, cluster 8). Of those, several are anti-apoptotic, such as the gene *BFAR,* an apoptosis regulator with anti-apoptotic activity.

In summary, the pattern of induction and repression of signaling genes in *C. albicans*-infected neutrophils revealed the involvement of major immunological pathways, most prominently NfκB and MAPK. This corresponds to the receptors and regulators also found to be affected, such as IL1-, complement- and TLR-mediated signaling. The presence of several DEGs involved in apoptosis, most of them inhibiting, indicates delay of apoptosis. In addition, the rearrangement of cytoskeleton and vesicular trafficking were reflected by numerous DEGs.

### Enrichment analyses reveal activation of the NOD-like receptor signaling pathway in *Candida*-triggered neutrophils

**Fig. 3:**
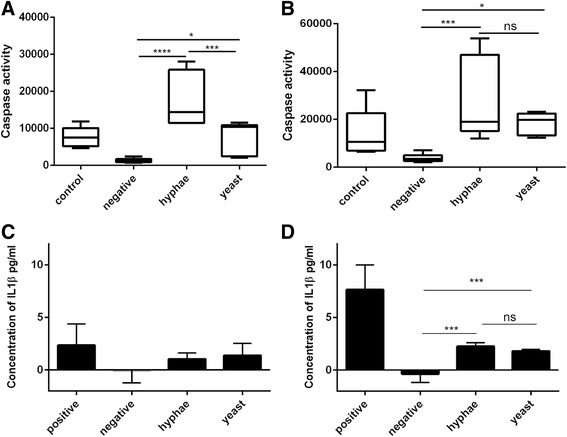
*C. albicans* triggers inflammasome activation in neutrophils quantified by Caspase-1 activation. Caspase-1 activity of *C. albicans* hyphae and yeast after challenging with human neutrophils for 1 h (**a**) and 3 h (**b**). As positive control neutrophils were stimulated with LPS (1 μg/ml), followed by ATP (5 mM, 1 h before adding the FLICA reagent). Amounts of IL-1β in supernatants of neutrophils challenged with *C. albicans* yeast or hyphae were quantified by ELISA after 3 h (**c**) and 6 h (**d**) of incubation. Shown are one representative experiment in triplicate for each assay from 2 experiments in total with very similar results, n = 2 (3). Statistical analysis was performed using a One-way ANOVA with Dunett’s post-test with * = *p* < 0.05, *** = *p* < 0.001 and **** = *p* < 0.0001

In addition to the functional description and analysis by fold-change of expression, we further compared the complete transcriptome of *C. albicans*-stimulated neutrophils to pathway databases for mammalian cells. The 318 DEGs identified were analyzed by ENRICHnet, KEGG pathway enrichment, and Gene Trail. As mentioned earlier, we have identified the gene *NLRP3* to be up-regulated in neutrophils. In line with this, the nuclear oligomerization domain (NOD)-like receptor signaling pathway was identified by all three approaches to be enriched. Of a pathway of 60 genes, seven were found within the 318 DEGs. These seven genes are *NLRP3*, *CXCL1*, *CXCL2*, *BIRC3*, *IL1B*, *IL8*, *RIPK2*. These seven genes are all up-regulated over the course of hypha and yeast infection over time, reaching their highest expression levels predominantly at the 60-min time point (Fig. 2). NOD and NOD-like receptors are intracellular PRRs that activate an inflammatory response. The NLRP subfamily is involved in inflammasome formation [48]. To gather further information on inflammasome activation, we analyzed caspase-1 activation in neutrophils infected with either *Candida* yeast or hyphae after one and three h of incubation (Fig. 3). In accordance with transcriptional data we observed caspase-1 activation by yeast and hypha infection. The latter resulted in stronger activation of caspase-1 than yeast infection after 1 h (Fig. 3a) and was similar in both after 3 h (Fig. 3b). Furthermore, we analyzed secretion of IL-1β, one of the major products of inflammasome activation. After three and 6 h of infection IL-1β concentration increased in supernatants reflecting caspase-1 activation (Fig. 3c and d). This finding supports transcriptional up-regulation of the *NLRP3* gene and provides further indications that *C. albicans* might trigger NLRP3-dependent activation of inflammasome-dependent pyroptosis in neutrophils.

### Neutrophils transcribe cytokine genes upon infection but not genes coding for archetype antimicrobial factors

**Fig. 4:**
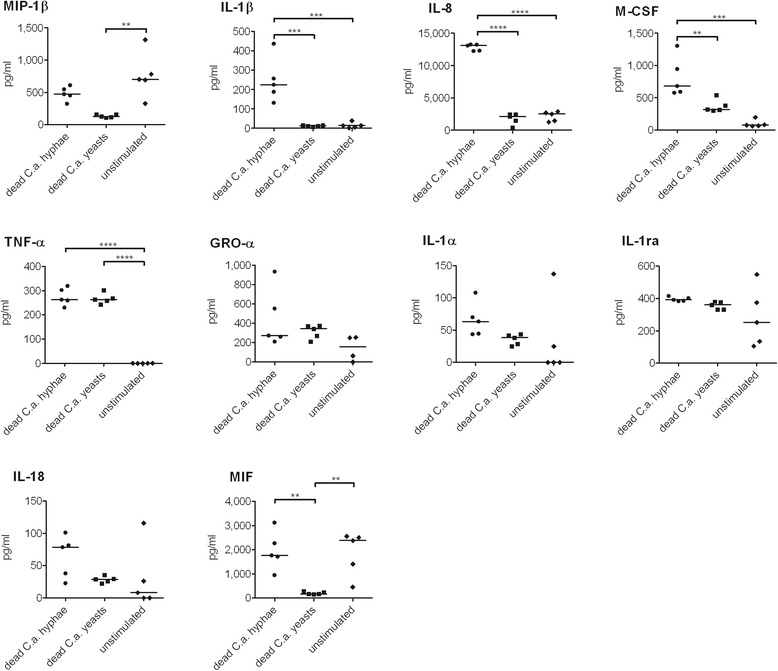
Cytokine secretion by neutrophils upon *C. albicans* infection. Neutrophils were stimulated either with thimerosal-killed *C. albicans* yeast, hyphae or left untreated. Supernatants of 18 h co-incubation were analyzed for cytokine release by Bio-Plex®. Each dot represent a value from an individual neutrophil donor, *n* = 5. Statistical analysis was performed using a One-way ANOVA with Bonferroni’s post-test with ** = *p* < 0.01, *** = *p* < 0.001 and **** = *p* < 0.0001

Interestingly, 16 cytokine genes were differentially regulated (Fig. 2d) with non-significant or marginal transcript levels shortly after infection but increasing transcript levels over time (Additional file 5: Figure S3, cluster 5 and 8). The cytokines coded by the up-regulated genes are for instance chemotactic to neutrophils, such as *CXCL1*, *CXCL2* and *IL8*. Further, *IL1A* and *IL1B* were also up-regulated whose products both bind to IL-1 receptors I and II. Neutrophil cytokine expression thus indicated orchestration of neutrophil recruitment and initiation of cross-talk with other immune cell types. To verify the role of neutrophils as cytokine producing cells, we infected freshly isolated neutrophils with dead *C. albicans* yeast and hypha cells for 18 h and quantified the resulting secretion of 10 cytokines with a Bio-Plex® approach from neutrophils of 5 individual donors (Table 2 and Fig. 4).

**Table 2:** Cytokine secretion of neutrophils infected with *C. albicans* yeasts and hyphae

Cytokine	Secretion	Gene	Expression (RPKM)			h60’/y60’	FC [log2]		DEG
			Control	60 min y	60 min h		60 min y	60 min h	
MIP-1β	UP-h	*CCL4*	54.4	171.8	194.5	1.1	2.20	2.35	yes
IL-1β	UP-h	*IL1B*	242.3	717.1	960.8	1.3	2.11	2.50	yes
IL-8	UP-h	*IL8*	9325.5	20,837.7	28,971.6	1.4	1.68	2.13	yes
M-CSF	UP-h	*CSF1*	10.6	11.8	13.6	1.2	0.68	0.86	no
TNFα	UP	*TNF*	30.6	39.6	30.6	0.8	0.90	0.50	no
Gro-α	n.s.	*CXCL1*	336.2	643.0	674.5	1.0	1.47	1.52	yes
IL-1α	n.s.	*IL1A*	2.6	33.3	37.7	1.1	4.24	4.39	yes
IL-1 ra	n.s.	*IL1RN*	225.0	486.0	524.3	1.1	1.65	1.74	yes
IL-18	n.s.	*IL18*	0.8	0.4	0.4	1.0	−0.34	−0.36	no
MIF	n.s.	*MIF*	0.8	0.4	0.3	0.7	−0.41	−0.91	no

Neutrophils were infected with dead *C. albicans* yeast or hypha cells and secreted cytokines were detected by Bio-Plex® at 18 h *p.i.*. Cytokines were either induced similarly with both (UP), secreted more in the presence of hyphae (UP-h), or not secreted (n.s.). Cytokine gene transcript levels (by RPKM) were analyzed after 60 min of stimulation, transcript levels in the infection with yeast and hyphae were related (h60’/y60’). Cytokine gene fold changes of expression (FC) are displayed after 60 min of cocultivation of neutrophils with *C. albicans* yeasts or hyphae. Statistically significant DEGs are pointed out

Among the 5 cytokines secreted in a *Candida*-dependent manner, we identified 4 cytokines that were secreted in higher concentrations in the cocultivation with hyphae than with yeasts: MIP-1β, M-CSF, IL-1β, and IL-8. Consistent with this finding, the RPKM values of the coding genes for these cytokines were slightly higher at 60 min *p.i.* when neutrophils encountered *C. albicans* hyphae, but not the yeast form (Table 2). In addition, the secretion of TNF-α was induced equally by both *Candida* morphotypes, while in this case the RPKM values were slightly higher in the yeast infection (Table 2). However, we could not detect inducible secretion of Gro-α, IL-18, IL-1α, IL-1ra, and MIF. Similar trends as with dead hyphae were observed when neutrophils were infected with live *C. albicans* yeast that form hyphae during the experiment. (Additional file 7: Figure S4). Of note, the differential expression of these 10 cytokine genes until 60 min *p.i.* was not entirely reflected by the ELISA-based cytokine secretion assay 18 h *p.i.* (Table 2). This indicates transcription of certain cytokine genes, secretion of pre-stored cytokines or shorter half-life of other cytokines. The overlap of transcript- and ELISA-based cytokine analyses confirms high reproducibility of our methods, despite possible donor variations of primary neutrophils.

In agreement with earlier investigations, most genes coding for typical neutrophil effector molecules, known to be formed during granulopoiesis, were not differentially expressed under tested conditions [11, 33]. However, analysis of the average transcription level, reflected by the RPKM value, revealed considerable variation between different effector molecules (Table 3) ranging from nearly no transcripts (e.g. defensin genes), via low and intermediate number of transcripts (e.g. cathelicidin gene *CAMP*) to higher levels for some genes coding factors related to the NADPH oxidase complex. Among the classical antimicrobials, the genes coding for the protein complex calprotectin (*S100A8* and *S100A9*) stood out. With constitutively high transcript levels showing RPKM values of 4721 and 17,890 in average those genes correspond to 1.39% and 5.25% of all RPKM values in uninfected and infected neutrophils, respectively. In other words, more than 6.5% of the entire neutrophil production of transcripts is dedicated to these two genes. This is in good agreement with the notion that the S100A8/A9 complex constitutes the major cytoplasmic protein of neutrophils [29].

**Table 3:** Unaffected expression of neutrophil effectors during *C. albicans* infection

Gene	Protein	Average RPKM	± SD	Rated
*S100A9*	S100A9	17,890	3030	++++
*S100A8*	S100A8	4721	837	++++
*NCF2*	NADPH oxidase, p67	669	103	+++
*LYZ*	lysozyme C	435	91	+++
*S100A12*	S100A12	372	78	+++
*NCF4*	NADPH oxidase, p40	293	40	+++
*MMP9*	gelatinase B	258	49	+++
*RAC2*	NADPH oxidase, Rac1 or Rac2	236	29	+++
*CYBA*	NADPH oxidase, p22-PHOX	115	19	++
*NCF1*	NADPH oxidase, p47	62	13	++
*CAT*	catalase	51	8	++
*RAC1*	NADPH oxidase, Rac1 or Rac2	25	5	+
*CAMP*	cathelicidin, LL-37	25	6	+
*CYBB*	NADPH oxidase, gp91-PHOX	16	3	+
*HMGB1*	high-mobility group protein 1/amphoterin	8	1	+
*TCN1*	transcobalamin-I	4	1	–
*CRISP3*	cysteine-rich secretory protein-3	1	0	–
*LTF*	lactotransferrin	1	0	–
*ELANE*	neutrophil elastase	0	0	–
*DEFA1B*	defensin 1	0	0	–
*DEFA1*	neutrophil defensin 1	0	0	–
*MMP8*	neutrophil collagenase	0	0	–
*CTSG*	cathepsin G	0	0	–
*MPO*	myeloperoxidase	0	0	–
*PRTN3*	proteinase 3	0	0	–
*BPI*	bactericidal/permeability-increasing protein	0	0	–
*DEFA3*	neutrophil defensin 3	0	0	–
*DEFA4*	neutrophil defensin 4	0	0	–
*DEFA5*	defensin 5	0	0	–
*DEFA6*	defensin 6	0	0	–

The transcript levels of typical neutrophil antimicrobial effector genes that were not differentially expressed during cocultivation with *C. albicans* was determined over all time points and conditions. Average and SD RPKM values are displayed and rated to illustrate the expression level. ++++: RPKM >1000; +++: RPKM ≥200; ++: RPKM ≥30; +: RPKM ≥5; −: RPKM ≥0

In summary, we confirmed that mature neutrophils have seized transcription of most antimicrobial factor genes. In contrast, numerous cytokines are transcriptionally induced during infection. This was additionally validated using protein quantification in supernatants of *C. albicans*-infected neutrophils.

### *C. albicans* response to neutrophils is dependent on morphotype and time point

For the in vitro infections analyzed later by RNA-Seq, we allowed *Candida* to adjust to the infection-related temperature and pH before addition of neutrophils for 30 min. The transcripts of these adjusted, but unchallenged fungal cells served as reference for DEG identification for each time point throughout the course of the infection. To have a comparable infectious load we adjusted the dry mass of *Candida* as described previously [39]. An average of 13.1 million reads was sequenced. Of those, 10.3 million reads were uniquely mapped (79%) and therefore utilized for gene expression analyses (Additional file 2: Table ST1). Only those *Candida* genes were considered as differentially expressed which changed transcript level higher than 4-fold at 15, 30 and 60 min neutrophil encounter compared to resting *Candida* cells (Additional file 8: Table ST4). Overall, the gene expression upon neutrophil encounter in *C. albicans* yeasts was altered more strongly than in hyphal cells. At time points 15 min, 30 min and 60 min *p.i.*, 258, 383, and 624 genes were differentially expressed in yeast cells compared to the reference, respectively. In hyphae, 73, 95, and 83 genes were found to be DEGs (Additional file 3: Figure S2B). Since our experimental conditions *per se* induce the switch from yeast to hypha, the difference between the numbers of DEGs in both morphotypes was not unexpected (Additional file 9: Figure S5C + D).

In contrast to the response by neutrophils, the overlap of differentially-regulated *Candida* genes throughout the entire infection was more abundant, but time point specificity could be observed as well (Additional file 3: Figure S2B, Additional file 9: Figure S5A + B). In general, more genes were involved in the response of *C. albicans* yeast towards neutrophils, but there is a considerable overlap between the yeast and the hyphal response (Additional file 9: Figure S5C + D).

**Fig. 5:**
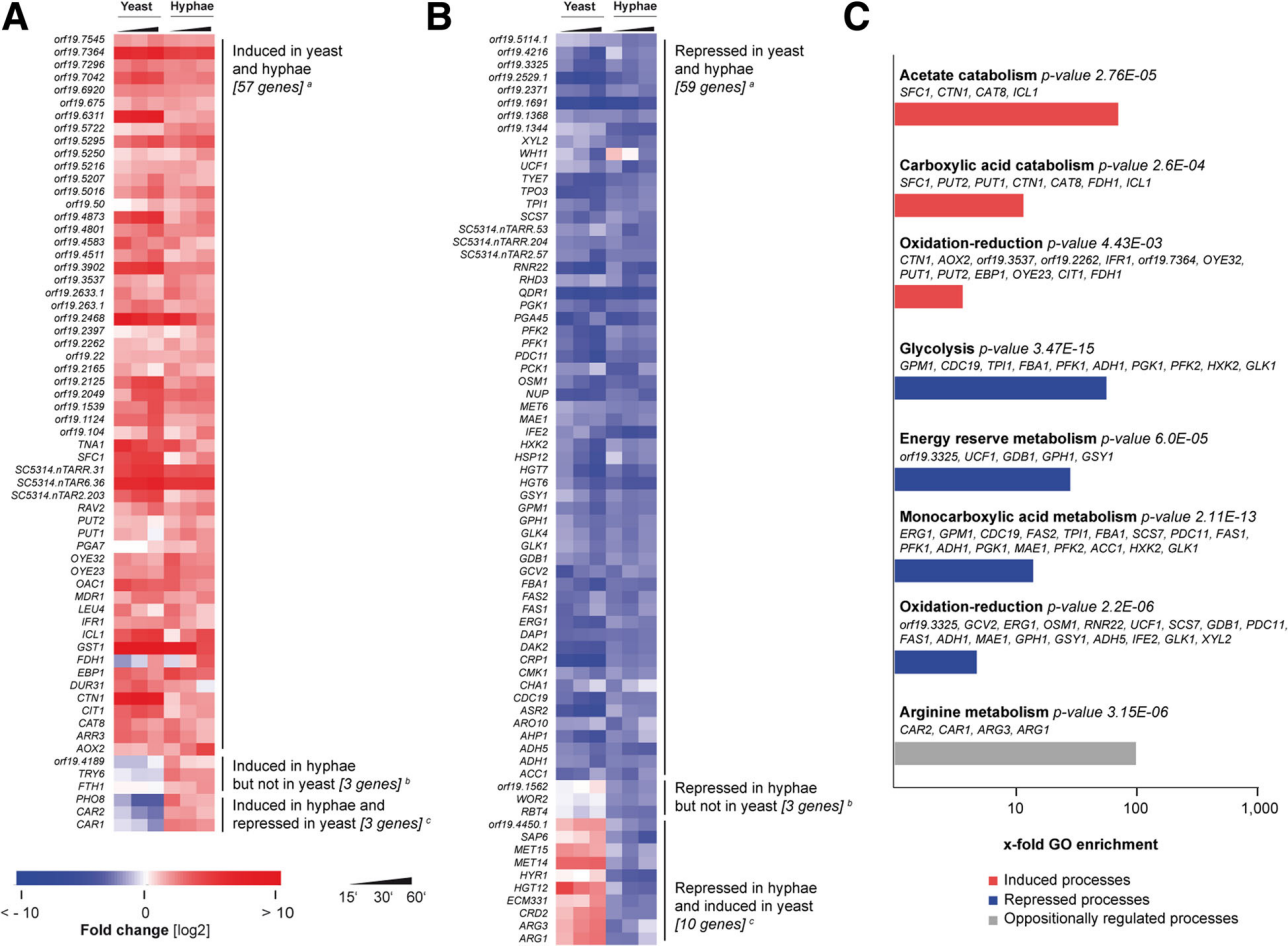
Core response of *C. albicans* during neutrophil infection. *Candida* genes differentially expressed in hyphae upon neutrophil encounter were considered as core response. Core DEGs were grouped according to their transcript profile at 15 min to 60 min *p.i*. and plotted for yeast and hyphae. **a** DEGs induced in hyphae, (**b**) DEGs repressed in hyphae. (A + B) Genes are called commonly repressed or induced in both morphotypes if in at least one time point in one morphotype a significantly differential expression was detected (≥ 4-fold) and in the other morphotype a regulation of ≥ 2-fold compared to reference was given at any time point (**a**). Genes are called induced or repressed in hyphae but not in yeast if in at least one time point in hyphae a significantly differential expression (≥ 4-fold) was detected and in yeast no regulation ≥ 2-fold was given at any time point (**b**). Genes are called induced or repressed in hyphae and repressed or induced in yeast if in at least one time point in hyphae a significantly differential expression (≥ 4-fold) was detected and in yeast significantly differential expression (≥ 4-fold) was given at any time point (**c**). **c** GO enrichment analysis of commonly (**a**) and not commonly induced or repressed genes (**b** and **c**). Corresponding sets of up- and down-regulated genes were mapped with the “GO term finder” at CGD (http://www.candidagenome.org/cgi-bin/GO/goTermFinder) to biological processes. Beside the assessment of the statistical significance of the gene set with a calculated *p*-value, the X-fold enrichment is calculated as ratio of percentages of the cluster frequency of tested gene set and the cluster frequency of genomic background. Red indicates up-regulation, blue indicates down-regulation. Samples from two independent experiments were analyzed, *n* = 2

For our analysis, we focused on the entity of genes differentially regulated in *C. albicans* hyphal cells upon neutrophil challenge (a total of 135 genes) and compared their level of regulation in yeasts and in hyphae during the infection. We refer to these 135 genes as the ‘*Candida* core response’ (not to be mistaken with the so-called ‘*Candida* core stress response’ by [49]). In doing so, we take into account that the in vitro infection conditions induce hyphal growth in *Candida* yeast cells and aim to focus on the general response of *Candida* towards human neutrophils (Fig. 5). A group of 57 genes was induced and 59 genes were repressed in both, yeast and hyphae (Fig. 5a and b). All genes described in the following were allocated to cellular functions using the *Candida* Genome Database and Kyoto Encyclopedia of Genes and Genomes - a GO-enrichment analysis was performed to identify the metabolic pathways affected in *C. albicans* upon neutrophil encounter (Fig. 5c).

### *C. albicans* alters primary sugar and amino acid metabolism upon neutrophil encounter and gene regulation is governed by few transcription factors

In agreement with earlier transcription studies using blood or phagocytes to trigger *Candida* yeast, our GO enrichment analysis revealed that the primary metabolism of *C. albicans* is strongly affected during neutrophil encounter [35–38]. We identified acetate catabolism, carboxylic acid catabolism, and oxidation-reduction as the three commonly induced processes (Fig. 5c). Because of the close relationship of these metabolic pathways, we found genes assigned to more than one. The induced genes *ICL1* and *FDH1* are each part of two of the three pathways. They code for enzymes involved in the glyoxylate cycle that has been shown to be induced upon phagocytosis in macrophages and in neutrophils [38, 50]. Thus, our RNA-Seq transcriptional analysis confirms relevant *C. albicans* pathways originally identified by microarrays. Among the oxidation-reduction genes induced, we found genes coding for different oxidoreductases and for enzymes involved in resistance against nitric oxide stress. The latter is in good agreement with a recent study revealing that nitric oxide stress resistance is essential for survival of *C. albicans* under neutrophil attack [50].

Similarly, four processes were revealed to be commonly repressed in neutrophil-triggered *Candida* cells: glycolysis, energy reserve metabolism, monocarboxylic acid metabolism, and oxidation-reduction (Fig. 5c). For instance, 10 genes of enzyme assigned to glycolysis were down-regulated. Thus, genes for enzymes functioning in oxidation-reduction have been both, up- and down-regulated. Yet, the oxidation-reduction DEGs do not overlap between the two modes of regulation. This points towards oxidative stress and a major remodeling of the metabolic profile of the *Candida* cells.

**Fig. 6:**
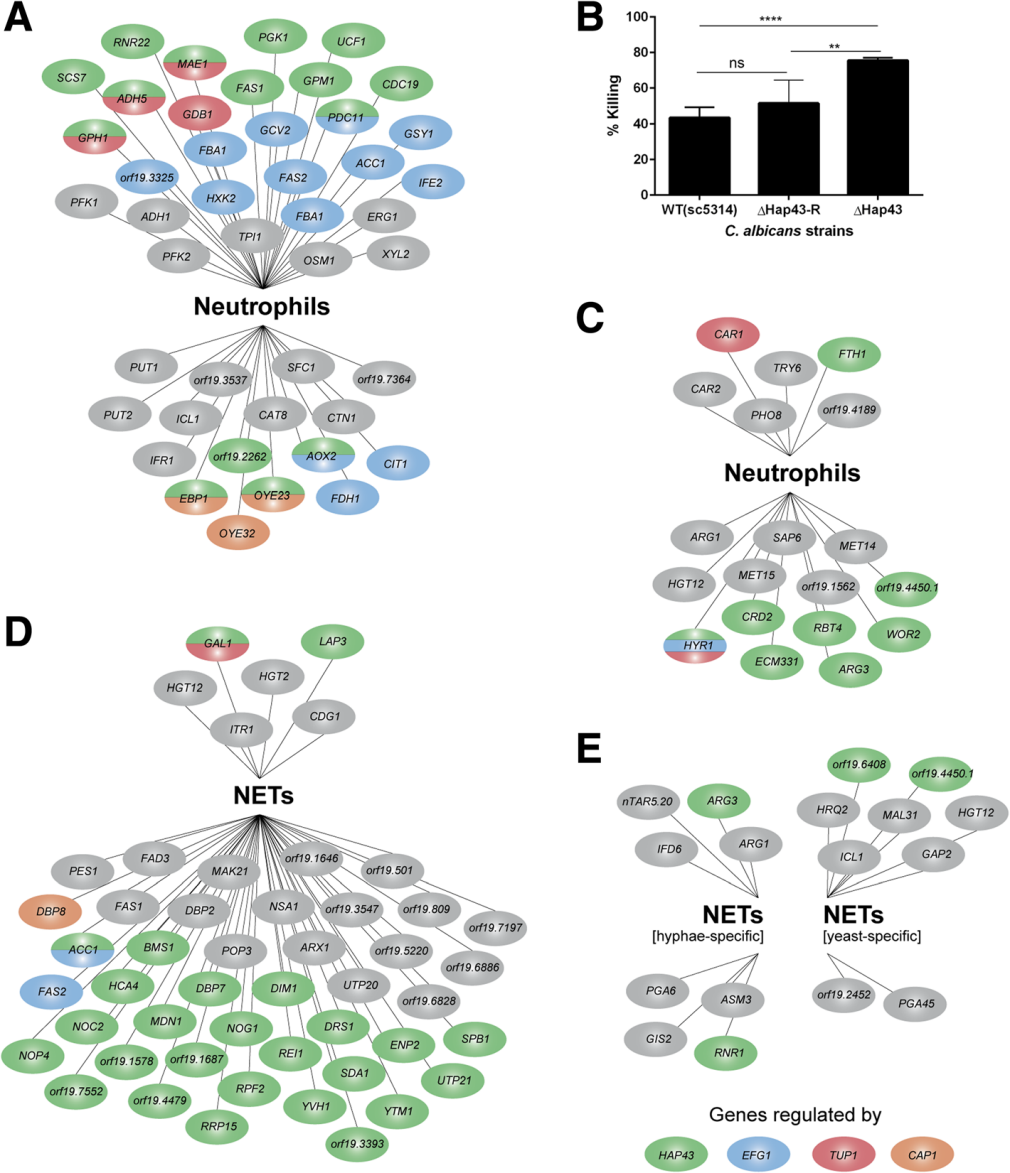
Transcriptional regulators controlling *C. albicans* DEGs during neutrophil/NET infection. DEGs of *C. albicans* during encounter of neutrophils or NET challenge were classified and visualized according to their control by transcriptional regulators, focusing on Efg1p (blue), Tup1p (red), Hap43p (green), and Cap1p (orange). **a** *Candida* genes from the GO enriched processes during neutrophil infection shown in Fig. 5c which were commonly induced or repressed; 28 induced genes in upper group, 16 repressed genes in lower group. **b** Percentage killing of *C. albicans* challenged for 1 h with human neutrophils (MOI 1). Viability of *C. albicans* was determined using a luciferase-based ATP assay. Shown is a representative experiment in triplicate from 2 experiments in total with very similar results, *n* = 2 (3). Statistical analysis was performed using a One-way ANOVA with Dunett’s post-test with ** = *p* < 0.01 and **** = *p* < 0.0001. **c** All morphology-dependently expressed *Candida* genes during neutrophil infection shown in in Fig. 5a and b; six hypha-induced genes in upper group, 13 hypha-repressed genes in lower group. **d** *Candida* genes from the GO enriched processes during NET challenge shown in Fig. 7c which were commonly induced or repressed; six induced genes in upper group, 43 repressed genes in lower group. **e** All morphology-dependently expressed *Candida* genes during NET challenge shown in Fig. 7a and b; four hypha-induced genes in upper left group, four hypha-repressed genes in lower left group, seven yeast-induced genes in upper right group, two yeast-repressed genes in lower right group

The majority of genes belonging to the aforementioned metabolic pathways, induced or repressed, are regulated by the transcription factors Efg1p, Tup1p, Cap1p, and Hap43p (Fig. 6a and c) in *C. albicans*. While Efg1p and Tup1p are regulators mainly involved in morphologic transition, Cap1p is a dedicated regulator of oxidative stress response [51, 52]. Hap43p was shown to be essential for iron homeostasis in *C. albicans* during infection [53, 54].

Since many DEGs identified in this study are regulated by Hap43p, we tested whether Hap43p-deficient strains were more susceptible to neutrophil killing than wild-type strains. For this purpose we infected neutrophils with *C. albicans* SC5314 wild-type strain, a *ΔHAP43* mutant strain, and a complemented strain derived from *ΔHAP43*. *Candida* viability was then quantified using an ATP-quantification assay. Indeed, the *ΔHAP43* mutant was significantly more susceptible to neutrophil-mediated killing than wild-type and complemented strains. Killing nearly doubled from 43% to 75% (Fig. 6b) confirming that gene regulation governed by Hap43p is indeed essential for *C. albicans* survival upon neutrophil encounter.

Cluster-wise analysis revealed that most pathways are commonly induced or repressed, whereas certain pathways were differentially regulated in yeast and hyphal cells challenged by neutrophils (Fig. 5a and b). In yeast, induction of the arginine and sulfur metabolism occurred, indicating high levels of oxidative stress, and re-arrangement of virulence factors (Fig. 5b). Remarkably, the arginine metabolism was oppositionally regulated in yeast and hyphae. While *ARG1* and *ARG3* were induced in yeasts and repressed in hyphae, *CAR1* and *CAR2* were induced in hyphae and repressed in yeasts (Fig. 5c and Additional file 10: Table ST5). In agreement, arginine biosynthesis, more precisely the genes *ARG1* and *ARG3*, were shown earlier to be induced in macrophage-engulfed *Candida* upon oxidative burst [55].

### ROS detoxifying enzyme genes are induced in yeast but not in hyphae upon neutrophil challenge

*C. albicans* fights ROS-mediated attacks by neutrophils with a variety of detoxifying enzymes. Of the 6 superoxide dismutases, Sod4p, Sod5p, and Sod6p are associated to the cell surface and Sod4p and Sod5p are crucial during interaction with phagocytes [50, 56]. Additionally, *C. albicans* has a catalase Cat1p and small redox proteins like thioredoxin. Remarkably, none of those genes was part of the ‘*Candida* core response’. A cut-off of 4-fold and a focus on DEGs in hyphae (‘*Candida* core response’) led to exclusion of the majority of ROS-detoxifying proteins. Nevertheless, *SOD3* and *SOD4* were induced more than 4-fold in yeast encountering intact neutrophils at one or more time points, but not in the hypha infection (Table 4). According to direct comparison of expression levels (by RPKM), hyphal cells already show high transcript levels before incubation with neutrophils (Additional file 11: Table ST6). This suggests that hyphal cells might already be better prepared for ROS-mediated stress. In summary, a number of *Candida* genes typically connected to virulence was indeed induced under cell-culture conditions in yeasts, but was concealed in hyphae, as most of the DEGs were already induced upon germination of hyphae.

**Table 4:** Transcription profile of selected *C. albicans* effectors genes upon neutrophil challenge

	*C. albicans* yeast [log2]				*C. albicans* hyphae [log2]			
	15 min	30 min	60 min	NETs	15 min	30 min	60 min	NETs
*SOD1*	−0.38	−0.02	−1.39	−0.41	1.15	0.72	0.24	0.94
*SOD2*	−0.60	−0.61	−0.62	0.12	−0.40	−0.66	−0.48	0.63
*SOD3*	1.75	−0.68	2.98	0.55	−0.24	0.30	1.19	1.04
*SOD4*	1.18	2.24	2.53	0.57	0.65	1.26	1.49	0.31
*SOD5*	1.45	0.90	0.43	1.64	0.91	0.83	0.56	0.15
*SOD6*	1.45	0.90	0.43	1.64	0.84	0.42	0.41	1.31
*CAT1*	0.67	−0.08	−1.08	1.33	1.00	−0.04	−0.87	1.20
*TRX1*	0.45	0.06	−0.97	0.56	1.19	0.61	0.28	0.83
*ECE1*	3.81	6.63	9.22	1.15	−1.16	−1.06	−1.48	−0.22
*PRA1*	3.32	1.03	1.49	1.77	−0.27	0.35	0.63	0.90
*ALS3*	1.56	3.05	4.52	−0.37	−1.13	−1.25	−1.62	−0.52
	Neutrophils				Neutrophils			

The transcript pattern of typical *C. albicans* effector genes affected during neutrophil encounter and NETs challenge is displayed by fold change. The transcript levels during neutrophil infection were corrected by the transcript levels in the same environment without neutrophils (medium, temperature) at every given time point

### NETs trigger similar transcriptional responses in *Candida* yeasts and hyphae and induce a broad stress response

In addition to analyzing the *C. albicans* response towards intact and viable neutrophils, we added yeasts or hyphae to NETs. Both morphotypes are known to be susceptible towards NETs [15]. For this in vitro set-up, NETs were generated from neutrophils using the abiotic stimulus phorbol myristate acetate (PMA). With this approach we ensured having nearly complete conversion of neutrophils to NETs [13]. Since NETs are a less dynamic stimulus than live neutrophils, we focused on one time point to compare to resting neutrophils as reference: 30 min *p.i.*. When *Candida* yeasts were added to NETs, transcript levels of 89 fungal genes were altered. Less genes - a total 61 DEGs - were affected in hyphae added to NETs (Additional file 3: Figure S2C, Additional file 8: Table ST4). Of the union of 116 DEGs, we identified 44 induced and 72 repressed DEGs which we considered for further analysis.

**Fig. 7:**
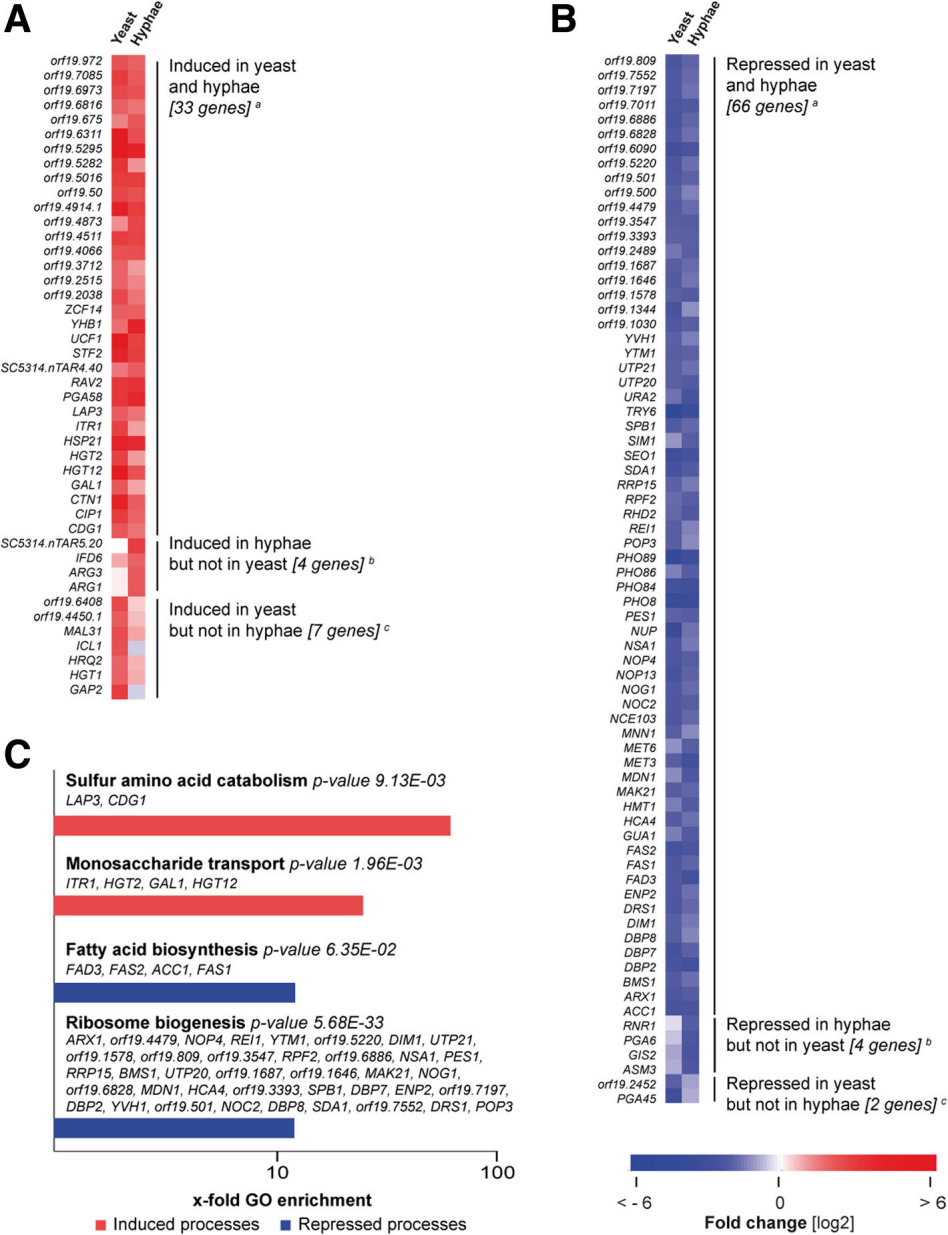
Transcriptional response of *C. albicans* challenged by NETs. *Candida* genes differentially expressed in yeasts and hyphae upon NET encounter were considered. DEGs were grouped according to their transcript profile at 15 min to 60 min *p.i.* in yeast and hypha (A + B): Genes are called commonly repressed or induced in both morphotypes if in at least one time point in one morphotype a significant differential expression was detected (≥ 4 fold) and in the other morphotype a regulation of ≥ 2-fold towards was given at any time point (**a**). Genes are called induced or repressed in hyphae but not in yeast (and *vice-a-versa*) if in at least one time point in hyphae or yeast a significant differential expression was detected (≥ 4-fold) and no regulation ≥ 2-fold was given at any time point for the other morphotype (**b** and **c**). **c** Corresponding sets of up- and down-regulated genes were mapped with the “GO term finder” at CGD (http://www.candidagenome.org/cgi-bin/GO/goTermFinder) to biological processes. Beside the assessment of the statistical significance of the gene set with a calculated p-value, the X-fold enrichment is calculated as ratio of percentages of the cluster frequency of tested gene set and the cluster frequency of genomic background. Red indicates up-regulation, blue indicates down-regulation. Samples from two independent experiments using different blood donors were analyzed, *n* = 2

The transcriptional response of *C. albicans* upon NET exposure indicated possible evasion mechanisms (Fig. 7a and b). Two cellular processes**,** sulfur amino acid metabolism and monosaccharide transport, were identified to be significantly up-regulated in *Candida* triggered by NETs, whereas fatty acid metabolism and ribosome biogenesis, were significantly repressed (Fig. 7c). Thus, this data points towards a solid stress response including nutrient uptake, oxidative stress detoxification, and strongly repressed protein expression.

Most DEGs were NET-specifically induced, with few exceptions. When comparing the ‘*Candida* core response’ of 135 DEGs (Fig. 5a and b) with the response towards NETs comprised of 116 genes (Fig. 7a and b), we found an overlap of only 24 shared genes. Similarly as in contact with intact neutrophils, the regulators Efg1p, Tup1p, Cap1p, and Hap43p were important for the *Candida* response as indicated by considerable regulation of a number of their gene targets (Fig. 6d).

### NET-Challenged *C. albicans* yeast induce sugar metabolism, hyphae induce arginine metabolism

To investigate a possible differential response of yeast and hyphae to NET attacks, we included both morphotypes of *C. albicans*. We found that the majority of genes to be affected in a similar fashion. Only a small proportion of the NET-induced DEGs genes were differentially regulated encountering NETs, namely 9 genes in yeasts and 8 genes in hyphae (Fig. 6e, Fig. 7a and b). Of note, NET challenge induced arginine metabolism (*ARG1* and *ARG3*) in hyphae, but not in yeast (Fig. 7a, Additional file 10: Table ST5). The opposite was found for the response to intact neutrophils, in which *C. albicans* yeast induced arginine metabolism whereas hyphae repressed it (Fig. 5b). Further, primary sugar metabolism was induced in yeasts (Fig. 7a).

## Discussion

Neutrophils are crucial for the defense against microbial invaders [1]. Although neutrophils have a short life span their life style is rather complex. In dependence of the milieu and the stimulus neutrophils launch a variety of different immune responses ranging from degranulation to phagocytosis and NET release. Notably, both *C. albicans* morphotypes are susceptible to neutrophils and NETs [15]. In the present study we therefore investigated the early interplay of human neutrophils with *C. albicans* yeasts and hyphae as well as responses of *Candida* yeasts and hyphae towards NETs by dual RNA-Seq.

The number of studies analyzing transcriptional responses in neutrophils is rather limited. The few studies conducted with different cytokines and microbes demonstrated that neutrophils react towards different stimuli with a distinct transcriptional program and a core gene response independent of the stimulus. A microarray-based comparison of neutrophil transcriptomes from LPS-, fMLF-, and *E. coli*-stimulated cells identified a mutual response that contained a high proportion of transcriptional regulators [32]. Overall, most of the DEGs in this study were assigned to transcriptional regulation and gene expression as well as signaling. Similarly, a core response and a stimulus-specific response were found when neutrophils were stimulated, ‘primed’, with TNF-α and GM-CSF and their transcriptomes were analyzed by RNA-Seq [34]. A comparable trend is reflected in our data. When analyzing the 318 DEGs in neutrophils encountering yeast and hypha *C. albicans*, we detected 36 hyphae-specific DEGs, thus 11% of the whole transcriptome were differentially expressed in the presence of this one stimulus. The experimental conditions chosen are known to induce *C. albicans* filamentation within a few hours, but are necessary to comfort neutrophils. Despite the short incubation time and minimized filamentation, we did not find any yeast-specific DEGs.

To find broader implications for signaling pathways engaged in neutrophils encountering *C. albicans* we performed a gene set enrichment analysis against publicly available expression array datasets. We revealed a significant over-representation of up-regulated genes associated with lipopolysaccharide-treated monocytes [57], from which the NOD-like pathway showed the most dominant overlap with our data. This suggests that similarly to monocytes during antibacterial responses, neutrophils trigger NOD-like intracellular receptor pathways upon recognition of fungal pathogen *C. albicans*. Of note, monocyte contamination of our neutrophil preparations were below 0.1% as determined by flow cytometry analysis, virtually excluding the possibility that similarities to monocyte expression profile stem from direct contaminations. Interestingly, NOD-like receptors are only present intracellularly where they detect microbes by their PAMPs or damage-associated molecular patterns (DAMPs). We detected the gene *NLRP3* to be induced similarly in the yeast and in the hypha response after 60 min: 3.6-fold and 3.4-fold, respectively. The only NOD-like receptor known to detect fungi is NLRP3, which leads to inflammasome activation [48]. To provide further indications for inflammasome activation in neutrophils upon *C. albicans* infection we performed caspase-1 activation assays and IL-1β release quantification using ELISA. Both assays showed induction upon yeast and hypha infection, with a significantly higher induction towards the pro-inflammatory, invasive hyphal form and thus supported our transcript analysis. Of note, one of the DEGs of the NOD-like pathway, *RIPK2*, is an established drug target for inhibiting this pathway with examples of clinically approved drugs including ponatinib and regorafenib inhibiting this kinase.

Moreover, our study supports the potential of neutrophils to be part of the cytokine-mediated orchestration at the *C. albicans* infection site. In agreement with earlier studies, we identified the cytokine genes *IL1A*, *IL1B*, and *IL8* to be induced in neutrophils after 1 h of stimulation with *C. albicans*, while the genes *TNF*, *IFNG*, *CSF2,* and *IL10* were not differentially regulated. Nevertheless, we detected the cytokine TNF-α in the supernatant of neutrophils after 18 h of coincubation with dead *C. albicans* indicating a delayed expression [33]. This finding was recently supported by Duggan et al. who also revealed TNF-α in the neutrophil supernatant after 4 h of cocultivation with live *C. albicans* and confirmed the secretion of MIP-1β, IL-8, as well as the lack of Gro-α and MIF in the neutrophil supernatants [58].

In contrast to transcriptional analyses in microbe-triggered neutrophils, there are a number of microarray-based studies investigating the transcriptional response of *C. albicans* to phagocytes such as neutrophils and macrophages/monocytes. The finding that *C. albicans* engulfed by macrophages alters its primary metabolism as a result of starvation by inducing the glyoxylate cycle [38], was followed by studies revealing that the metabolic response is accompanied by dramatic reprogramming on many levels: For instance, the induction of hyphal growth to enable the escape from macrophages, the activation of an oxidative stress response, DNA damage repair or arginine biosynthesis [37]. *C. albicans* engulfed by neutrophils was demonstrated to strongly induce amino acid metabolism [59]. Interestingly, this induction was dependent on the presence of serum and in addition stronger when serum was fresh and not heat-inactivated. This enhancement was most probably due to an increased phagocytosis rate. Simultaneously, studies with human blood as well as enriched neutrophils confirmed the important role of the glyoxylate cycle and the amino acid metabolism [35, 36]. A phagocyte can therefore be described as an environment rich in amino acids and poor in glucose [55]. In our study, we focused primarily on those genes that were differentially expressed in hyphae. In doing so, we chose to not discuss a number of genes that were shown to be strongly affected upon phagocyte encounter in previous studies. All aforementioned studies used *C. albicans* yeast with MOIs of 1–5 yeast cells per phagocyte to start the interaction between the fungus and the host cell. In our study, we used an MOI of 1 for yeast and a similar amount of hyphae quantified by dry mass correlation. Even though we did not include serum in our experimental set-up and only considered DEGs with an altered transcript level beyond 4-fold, there were striking similarities to earlier studies. For instance, *ICL1*, known to be induced upon challenge with blood, enriched neutrophils, macrophages, and upon engulfment by neutrophils, was also up-regulated in yeast encountering neutrophils in our experiment [35, 36, 38, 50]. Remarkably*, ICL1* was similarly up-regulated in yeast challenged by NETs, even though these trap-like structures cannot entirely enclose a fungal cell. Genes from the amino acid metabolism that were demonstrated to be induced in neutrophil-encountering yeast, e.g. *MET1*, *MET3, ARG1*, *ARG3*, *LEU1*, *LEU2, and LEU4* were also induced in our study [36, 59]. Interestingly, we revealed *ARG1* and *ARG3* representing two out of 4 genes to be regulated in opposite manner in the yeast and the hyphal response, i.e. they were down-regulated in hyphae-challenged with neutrophils, but up-regulated in yeast. The transcript of those two genes is described to be induced in *C. albicans* yeast upon phagocytosis and related to ROS [55, 59]. To our surprise, both genes were induced in NET-challenged *Candida* hyphae, but not in yeast. Since we could not detect high levels of ROS in our NET preparations (Additional file 12: Figure S6), this might point to the increased accumulation of intrinsic *C. albicans* oxidative stress in hyphae when challenged by NETs compared to intact neutrophils.

As main transcriptional regulators of the affected metabolic pathways in *C. albicans* encountering neutrophils we identified Efg1p, Tup1p, Cap1p, and Hap43p. A mutant strain deficient in Hap43p was significantly more susceptible to killing by neutrophils and therefore demonstrates that regulation of metabolic pathways in *C. albicans* by Hap43p was crucial for evasion of neutrophil-mediated killing.

In contrast to a number of earlier similar studies, we did not include serum. Certainly, antibody-mediated recognition and complement are important during the innate immune response against *C. albicans*. On the other hand, the composition of serum, e.g. the concentration of bacterial peptidoglycans, and therefore its impact on hyphae induction likely varies [60]. Further, we performed comparative killing assays in a previous study in which we did not see a significant difference between the *Candida*-killing capacity of human neutrophils in RPMI with and without 2% serum [61]. This is supported by a recent study demonstrating two distinct killing mechanisms of *C. albicans* by neutrophils, depending on opsonization [62]. In conclusion, we are convinced that an in vitro infection without serum is a simplified, but nevertheless valid approach to understand the fundaments of the neutrophil–*C. albicans* interaction. To account for the donor variability we included different donors of blood as neutrophil sources. Taken together, the overall similarities in the yeast response to neutrophils in our study indicate the comparability of our study with earlier studies and by this establish the ground for the new findings regarding the hyphae response to neutrophils and the *C. albicans* response to NETs.

## Conclusions

We dissect the interaction of *C. albicans* yeast and hyphae with intact neutrophils as well as NETs. The neutrophil response showed a major *Candida* morphotype-independent and a minor hyphae-specific response. Similarly, the *Candida* response towards both intact neutrophils and NETs was majorly morphotype-independent. The *Candida* response to NETs emerged to be distinct from the response to intact neutrophils. We provide indication that neutrophils considerably contribute to the orchestration of complex immunological responses upon encounter of *C. albicans* by i.e. inflammasome induction and release of numerous cytokines. *C. albicans* in turn sought to avoid these diverse neutrophil attacks by regulating required genes using the transcription factor Hap43p as a key regulator to evade killing by neutrophils. This knowledge can contribute to a more specific targeting of hyphal *Candida* as well as modulating the *Candida*-induced inflammatory response during infection.

## Methods

### Growth of *Candida albicans* and dry mass adjustment

Before use, *C. albicans* clinical isolate SC 5314 was re-cultivated from a frozen glycerol stock on YPD plates for 24 h at 30 °C and inoculated to an overnight YPD medium culture (1% yeast extract, 2% Bacto peptone and 2% glucose) at 30 °C with continuous shaking. To harvest yeast cells, a subculture in YPD was inoculated with OD_600_ 0.1 and incubated at identical conditions for 3 h. To harvest hyphae, a subculture was inoculated at a start OD_600_ 0.1 in RPMI1640 without phenol red (w/o PR) and incubated at 37 °C with vigorous shaking. Yeast cells and hyphae were harvested by centrifugation at 3000×g for 5 min in a pre-warmed centrifuge and washed 3 times with pre-warmed RPMI *prior* to the experiments. Cell numbers for *Candida* yeast were calculated by OD_600_ – CFU correlation (*C. albicans*: OD_600_ 1 ≙ 2.9 × 10^7^ CFUs/ml). Using an XTT / dry mass correlation, the dry mass of yeast and hyphae per milliliter were adjusted before infection [39].

### Isolation of human neutrophils

Neutrophils were isolated from venous blood of healthy volunteers as previously described [13]. Briefly, blood was layered 1:1 on Histopaque-1119 and separated by centrifugation for 20 min at 800×g. Granulocyte-rich buffy coats were collected and cells washed with PBS with 0.5% (*w*/*V*) human serum albumin (HSA). Cells were segregated on a discontinuous phosphate-buffered Percoll gradient: 65%, 70%, 75%, 80%, and 85% by centrifugation for 20 min at 800 x g. The cell layer formed at the 80% to 75% interface was collected and washed as before. *Prior* to use, neutrophils were counted, assessed for viability with trypan blue staining and diluted to desired concentration in RPMI1640 without phenol red (w/o PR). All assays were performed in RPMI1640 w/o PR, if not stated otherwise.

### Flow cytometry analysis of neutrophil suspension after isolation

To judge neutrophil purity of the isolation protocol, we analyzed cell suspensions from 5 independent isolations with flow cytometry. Staining was performed according to standard protocols with antibodies against human CD3-FITC, CD14-APC, CD19-PE, MHC class II (HLA-DR,DP,DQ)-FITC and CD11b-APC (eBioscience or BD Biosciences). Non-specific binding was blocked by 15 min pre-incubation in PBS with 2%(V/V) fetal calf serum (FCS). Peripheral blood monocytes (PBMCs) were used as positive staining controls. Data was recorded on a FACSCantoII™ and analyzed using the FACSDiva™ software (BD Biosciences).

### Preparation of NETs

Neutrophils (3 × 10^6^) were seeded into a 24-well plate. NET formation was induced as described before [13]. Briefly, neutrophils were stimulated with 100 nM phorbol myristate acetate (PMA) and incubated for 4 h at 37 °C with 5% CO_2_. Activation and conversion of neutrophils was controlled microscopically. After 4 h, supernatant was gently removed from NETs and 200 μl pre-warmed RPMI1640 w/o PR added.

### Infection of neutrophils or NETs with *Candida albicans*

Subcultered *C. albicans* was washed in pre-warmed RPMI1640 w/o PR in a pre-warmed centrifuge to allow *Candida* to adjust to temperature and pH. Fungal yeast and hyphae were diluted according to an XTT/dry mass correlation to ca. Four hundred ninety microgram per milliliter [39]. Of these, 150 μl were seeded into a 24-well plate, which equals 74 μg. This dry mass corresponds to 3 × 10^6^ yeast cells from a 3 h subculture in YPD and thus to MOI of 1 in a yeast infection. Neutrophils were diluted to 1.5 × 10^7^ cells/ml and 200 μl added to 24-well plates to start infection. To support contact of neutrophils and *Candida* cells, 24-well plates were centrifuged for 5 min at 300×g in a pre-warmed centrifuge. Incubation occurred for 15 min, 30 min and 60 min at 37 °C with 5% CO_2_. An incubation of 30 to 60 min was shown before to allow a substantial proportion of neutrophils to interact with and engulf *C. albicans* yeast [36, 61]. NETs were infected in a similar manner, but the infection was only allowed for 30 min. For each time point, *C. albicans* controls w/o neutrophils were included. Unstimulated neutrophils kept in RPMI1640 w/o PR served as a control. After the incubation, 24-well plates were centrifuged for 5 min at 300 x g in a pre-warmed centrifuge. For RNA isolation supernatant was carefully and quickly removed and discarded and 600 μl RLTplus including 1% (V/V) β-mercaptoethanol (β-ME) were added, and cell residues were recovered from wells by vigorous scratching and repetitive pipetting. For wells dedicated to *C. albicans* RNA isolation, 3.5 μl β-ME were added to reach 1% (V/V). Similarly, cell residues were recovered from the wells by vigorous scratching and repetitive pipetting. The resulting suspension was shock-frozen by dropping it into liquid nitrogen. Suspension ‘pearls’ were kept at −80 °C until proceeding with the RNA isolation. All infections were performed in duplicates to facilitate the parallel isolation of RNA from *C. albicans* and neutrophils.

### RNA isolation

Disruption of frozen suspension pearls was carried out using Mixer Mill MM 200 (RETSCH, Germany) with 30/s shaking frequency under cryogenic conditions. The powder was resuspended in lysis buffer RLTplus (QIAGEN, Germany), supplemented with 1% (V/V) of β-ME. Extraction of total RNA was performed according to QIAGEN’s Mechanical Disruption Protocol for total RNA isolation from yeast, using the RNeasy Plus Mini Kit. The experiments were performed in duplicates. RNA quality and quantity was evaluated using an Agilent 2100 Bioanalyzer with RNA 6000 Nano or Pico Chips.

### cDNA library preparation for Illumina sequencing

cDNA libraries were prepared with 200 ng of total RNA and Illumina’s TruSeq RNA Sample Preparation v2 protocol. Sequencing was performed using an Illumina HiSeq2000. To assess concentration and ensure appropriate size distribution (between 200 and 570 bp) cDNA libraries were checked using Bioanalyzer DNA 1000 chips. Sequencing run was carried out with single-end 50 bp long reads following manufacturer’s instructions with average sequencing depth of 35 and 12 million reads for neutrophils and for *Candida* transcriptome, respectively.

### Mapping and quantification of RNA-Seq data and DEG identification

Reads were mapped against the human reference genome [hg19] and *C. albicans* reference genome Assembly 21 using NextGenMap (v. 0.4.12) with default settings [63]. A summary of all analyzed samples for RNA-Seq including sequencing depth and mapping statistics is listed in Additional file 2: Table ST1. Downstream quantification (rpkmforgenes.py, http://sandberg.cmb.ki.se/rnaseq/) of genes in raw read counts as well as RPKM (= reads per kilobase of exon model per million mapped reads according to Mortazavi et al. [64]) was carried out exclusively with uniquely mappable reads using gencode annotation v17 and annotation provided by Grumaz et al. [65] for human and for *C. albicans*, respectively (Additional file 11: Table ST6). To identify differentially expressed genes (DEGs) between two conditions, we applied edgeR (version 3.4.2) with two biological replicates per condition based on raw read counts [66]. In order to reduce background signals, we applied stringent criteria considering only genes with an adjusted *p*-value (FDR) < 0.05, a mean log2 CPM (counts per million) > 4 and a log2 fold change ≤ −2 or ≥ 2 for *Candida* genes or a log2 fold change ≤ −1.5 or ≥ 1.5 for human genes as being significantly differentially regulated between two conditions.

### Enrichment analysis of neutrophil DEGs

For the enrichment analysis, we focused on 318 DEGs which represent genes that were differentially expressed (up- or down-regulated) with a log2 fold change of ≥ 2 and a *p*-value of < 0.05 in at least one time point upon yeast or hyphae infection. To minimize bias inherent in a single analysis approach we subjected this list to enrichment analyses using three web-based tools, ENRICHnet (http://www.enrichnet.org/), KEGG pathway analysis (https://david.ncifcrf.gov/) and GeneTrail (http://genetrail.bioinf.uni-sb.de/). The ENRICHnet analysis was undertaken against the STRING database (a database of functional protein-protein interactions - https://string-db.org/) as a pre-step to a KEGG pathway analysis. Both steps form part of the ENRICHnet analysis pipeline and the output file is ranked according to statistical significance as determine using a Fisher’s t test. Only one pathway was significant by this measure and this was the NOD-like receptor signaling pathway with a *p* value of < 0.05. By contrast KEGG pathway analysis using the DAVID web tool (https://david.ncifcrf.gov/) simply assesses the statistical significance of sets of genes based on over-representation between a numerically matched random background geneset. By these criteria the NOD-like receptor signaling pathway was still the most significant with a *p* value of < 0.05 (again based on a Fisher’s t test) however two other pathways were also significant based on this threshold – MAPK signaling and cytokine-cytokine receptor interaction. GeneTrail was used with a multiple testing correction (Benjamin & Hochberg 1995) to identify significant KEGG pathways based on an FDR of < 0.05. This yielded four pathways significant at < 0.05 which were in ranked order of significance MAPK signaling, cytokine-cytokine receptor interactions, NOD-like receptor signaling pathway and the hematopoietic cell lineage.

### ROS production

ROS production was measured using a luminol-based chemiluminescence assay. NETs from 5 × 10^4^ neutrophils were supplemented with 50 μM luminol (Sigma-Aldrich) and 1.2 U/ml horseradish peroxidase (Sigma-Aldrich) were seeded in a white 96-well plate (Nunc). NETs were infected with *C. albicans* at MOI 1 and 2 or left uninfected. As reference, intact neutrophils were infected similarly or stimulated with 100 nM PMA (Sigma-Aldrich). The chemiluminescent signal was detected with an Infinite 200 luminometer (Tecan Nordic, Mölndal, Sweden) every 3 min for 6 h in 3 replicates. Cells were kept at 37 °C with 5 % CO_2_ during measurements.

### *Candida albicans* Cultivation for bio-Plex® cytokine quantification

*Candida albicans* clinical isolate SC5314 was cultured overnight in YPD at 30 °C on a shaker. Cell numbers were calculated by OD_600_ correlation (*C. albicans*: 1 OD_600_ = 2.9 × 10^7^ cells/ml). Hyphal growth was induced by culturing *C. albicans* (starting OD_600_ 0.1) in RPMI 1640) at 37 °C and yeast growth was induced in YPD at 30 °C for 3 h on a shaker. Subsequently *C. albicans* hyphae and yeast were killed by adding 1% (w/V) thimerosal (Sigma-Aldrich) and incubating protected from light overnight on a roll shaker. Cells were washed 3 times in 50 ml PBS prior to cytokine experiments. A plating control of washed, dead *C. albicans* hyphae and yeast revealed no viable colonies after incubating for 3 d at 30 °C.

### Stimulation and suspension cytokine array analysis

Neutrophils were seeded in sterile 96-well plates at a density of 5 × 10^5^ cells per well in a final volume of 100 μl RPMI. Cells were stimulated with dead *C. albicans* hyphae or yeast at MOI 2 or left untreated for 18 h at 37 °C and 5% CO_2_. After incubation cells were spun down, supernatants were collected and subsequently stored at −80 °C for analysis of cytokine release.

Following cytokines were measured with a Bio-Plex® Pro human cytokine 24–Plex (Group II) and 27–Plex (Group I; both Bio-Rad, Hercules, USA): We selected the following cytokines from group I: IL-1β, IL-1ra, IL-8, MIP-1β, TNF-α and group II**:** IL-1α, IL-18, GRO-α, M-CSF, MIF; The Bio-Plex® analysis was performed using a Bio-Plex® 200 System with Bio-Plex Manager 4.1.1 (Bio-Rad) software. We compared cytokine levels in supernatants of hypha-stimulated and yeast-stimulated neutrophils for Gro α, IL-1α, IL-1β, IL-1ra, IL-8, IL-18, M-CSF, MIF, MIP-1β and TNF α. One way ANOVA analyses with Bonferroni’s posttest were performed using Graphpad Prism Software 5.03. Samples were considered being significantly different with *p* < 0.05.

### Caspase-1 assay to quantify inflammasome activation

Activation of caspase-1 was investigated using a FAM FLICA™ Caspase-1 Assay Kit (Immunochemistry, Bloomington, IN, USA). Neutrophils were seeded at 1 × 10^5^ cells per well in 96-well microplates and infected with *C. albicans* at MOI 1 for 1 h and 3 h. Afterwards, cells were labeled with FLICA (FAM-YVAD-fmk) according to manufacturer’s instructions. As positive control served neutrophils + LPS (1 μg/ml), followed by addition of ATP (5 mM, 1 h before adding the FLICA reagent). Cells were analyzed in a plate reader (FLUOstar omega, BMG labtech, Ortenberg, Germany) to measure caspase-1 activity.

### Quantification of IL-1β in supernatants of stimulated neutrophils

Neutrophils were seeded at 1 × 10^5^ cells per well in 96-well microplates and infected with *C. albicans* at MOI 1 for 3 h and 6 h in quadruplicate. Cell-free culture supernatants were harvested and IL-1β concentration was measured by ELISA (Biolegend-ELISA MAX™ 9727 Pacific Heights Blvd, San Diego, CA, USA), according to manufacturer’s instructions. As positive control served neutrophils + LPS (1 μg/ml). Sensitivity or minimum detectable concentration of IL-1β was 0.5 pg/ml. For the assay, a standard recombinant cytokine preparation was used to estimate cytokine concentrations in samples.

### *C. albicans* cell viability assay

*C. albicans* WT SC5314, CaHap43D2–1–8-1, CaHap43R3–1–5-2, CaHap43D1–1–9-1 strains were used [53]. Viability was assessed by ATP quantification using luminescent CellTiter-Glo Promega kit. The fungal strains were (1 × 10^5^ cells/well) were challenged with neutrophils (1 × 10^5^ cells/well) at MOI 1. The plates were incubated at 37 °C for 1 h. Neutrophils were lyzed with Triton X-100. After 15 min incubation plates were washed with PBS. Luminescence signals were recorded using a Tecan Infinite F200 microplate reader.

## Additional files

Additional file 1: Figure S1. Purity analysis of neutrophil isolation. Cellular composition after Percoll gradient purification (A). Neutrophils (CD11b^+^MHCII^−^): 91.8%, monocytes (CD14^+^): 0%, T cells (CD3^+^): 0.7%, DC (CD11c+): 0.1%, B cells (CD19+): 0%, other cells (predominantly FSC^int^ SSC^Hi^ eosinophils): 7.4%. Three multi-color staining panels were used: CD3-FITC, CD19-PE and CD14-APC to distinguish T- and B-cells as well as monocytes; HLA-DP/DQ/DR-FITC and CD11b-Pacific Blue™ for neutrophils and CD11b-Pacific Blue™, CD11c-FITC for DCs. FACS blots of gating strategies used (B). Results from one representative of 5 independent experiments shown. (TIFF 1320 kb)

Additional file 2: Table ST1. Mapping results. Mapping statistics of neutrophil and *Candida* reads using NextGenMap. (XLSX 41 kb)

Additional file 3: Figure S2. Overview of DEGs over time. The number of DEGs in neutrophils during *C. albicans* infection (A), in PMN-treated *C. albicans* cells (B) and in NET-treated *C. albicans* cells (C) over time. Red indicates up-regulation, blue indicates down-regulation. (TIFF 206 kb)

Additional file 4: Table ST2 A-C. Differential gene expression analysis of neutrophils. Comprehensive gene expression analysis table of neutrophils infected with yeast (A) and hyphae (B). Each condition was tested against the uninfected PMN control. Union of all 318 protein-coding DEGs in neutrophils (C). (XLSX 19148 kb)

Additional file 5: Figure S3. Clustering of 318 DEGs in neutrophils infected with *C. albicans*. The entity of protein-coding DEGs in neutrophils affected during a *C. albicans* infection was clustered via QT-Clustering using Mayday based on their fold changes over the time which were z-score normalized for better visualization purposes. The cluster profile hallmarks are indicated. (TIFF 728 kb)

Additional file 6: Table ST3. Most altered DEGs in neutrophils infected with *C. albicans*. Up- and down-regulated DEGs of neutrophils infected with *C. albicans* yeast and hyphae were sorted by their respective fold change of expression. Positive numbers indicate the ranking amongst up-regulated DEGs; negative numbers indicate the ranking amongst the down-regulated numbers (PDF 52 kb)

Additional file 7: Figure S4. Cytokine secretion by neutrophils upon *C. albicans* infection. Neutrophils were analyzed for cytokine release upon 18 h stimulation with thiomersal-killed *C. albicans* hyphae or live *C. albicans* (initially yeast). None of the analyzed cytokines showed a statistically significant difference between stimulation with dead hyphae or live *C. albicans*, indicating that dead hyphae evoke similar responses in neutrophils. Statistical analysis was performed by using a One-way ANOVA with Bonferroni’s post-test (*n* = 5). (TIFF 724 kb)

Additional file 8: Table ST4 A-C. Differential gene expression analysis of *Candida*. Comprehensive gene expression analysis table of neutrophil-or NET-treated *Candida* cells in yeast form (A) and in hyphae form (B). Respectively to the form, each condition was tested against the unchallenged, but adjusted fungal cell controls. Union of all 797 DEGs in *Candida* cells (C). (XLSX 4321 kb)

Additional file 9: Figure S5. Overlaps of DEGs in yeast and hypha *C. albicans* challenged with neutrophils. Overlaps of DEGs in *C. albicans* (A) yeast and (B) hyphae challenged with neutrophils throughout the time course. Overlap of morphotype-specific *Candida* response of (C) induced and (D) repressed DEGs. Samples from two independent experiments using different blood donors were analyzed, *n* = 2. (TIFF 353 kb)

Additional file 10: Table ST5. Differential regulation of arginine metabolism genes in *C. albicans*. DEGs involved in arginine metabolism of yeast and hypha *C. albicans* infecting neutrophils and NETs are displayed. The transcript level is indicated by fold change (log2). (PDF 22 kb)

Additional file 11: Table ST6 A-D. Quantification results. Comprehensive gene quantification table of neutrophil transcriptome in read counts (A) and in RPKM (B) as well as of *Candida* transcriptome in read counts (C) and in RPKM (D). (XLSX 18944 kb)

Additional file 12: Figure S6. ROS levels in NET vicinity. ROS produced by in vitro released NETs and neutrophils (PMNs) were quantified by a luminol-based assay over 6 h to test for background ROS due to NET preparation in comparison to stimulated PMNs (A + B). (A): ROS in vicinity of unstimulated, PMA-stimulated, or *Candida*-infected NETs; (B) ROS in vicinity of unstimulated, PMA-stimulated, or *Candida*-infected PMNs. Averages and SD plotted of 3 replicates, *n* = 3. (TIFF 761 kb)

## Abbreviations

ATP: Adenosine triphosphate
Ca: *Candida*
Caspase: Cysteine aspartic acid specific protease
CD: Cluster of differentiation
DEG: Differentially expressed gene
ELISA: Enzyme-linked immunosorbent assay
FACS: Fluorescence-activated cell sorting
FLICA: Fluorochrome inhibitor of caspases
fMLF: formyl-Met-Leu-Phe
GI: Gastrointestinal tract
LPS: Lipopolysaccharide
ME: β-mercaptoethanol
MOI: Multiplicity of infection
NADPH: Nicotinamide adenine dinucleotide phosphate
NET: Neutrophil extracellular trap
NOD: Nuclear oligomerization domain
*p.i.*: *Post infection*
PAMP: Pathogen-associated molecular pattern
PBS: Phosphate-buffered saline
PMA: Phorbol-12-myristate-13-acetate
PRR: Pattern recognition receptor
RNA-Seq: RNA identification and quantitation via next generation sequencing
ROS: Reactive oxygen species
RPKM: Reads Per Kilobase Million
RPMI: Roswell Park Memorial Institute medium
TLR: Toll-like receptor
WT: Wild type
YPD: Yeast peptone dextran

## Glossary

Neutrophil core response: Entity of neutrophil genes differentially expressed at one or more time points upon infection with *C. albicans* yeast and hyphal cells.
*Candida* core response: Entity of *C. albicans* genes differentially expressed in hyphal cells upon neutrophil encounter at one or more time points.
*Candida* core stress response: Entity of *C. albicans* genes differentially expressed upon different abiotic stressors [49].
