# Correction to: Dual transcriptome of the immediate neutrophil and *Candida albicans* interplay

**DOI:** 10.1186/s12864-017-4207-3

**Published:** 2017-11-13

**Authors:** Maria J. Niemiec, Christian Grumaz, David Ermert, Christiane Desel, Madhu Shankar, José Pedro Lopes, Ian G. Mills, Philip Stevens, Kai Sohn, Constantin F. Urban

**Affiliations:** 1 Department of Clinical Microbiology, Umeå Centre for Microbial Research (UCMR) & Laboratory of Molecular Infection Medicine Sweden (MIMS), Umeå University, Umea, Sweden; 2 Fraunhofer Institute for Interfacial Engineering and Biotechnology IGB, Stuttgart, Germany; 3 Institute of Clinical Microbiology, Immunology and Hygiene, University Hospital Erlangen, Friedrich-Alexander-Universität Erlangen-Nürnberg, Erlangen, Germany; 4 Prostate Cancer Research Group, Center of Molecular Medicine Norway (NCMM), Oslo, Norway; 5 Department of Molecular Oncology, Institute of Cancer Research, Radium Hospital, Oslo, Norway; 6 PCUK/Movember Centre of Excellence for Prostate Cancer Research, Centre for Cancer Research and Cell Biology (CCRCB), Queen’s University Belfast, Belfast, UK; 7 University of Stuttgart IGVP, Stuttgart, Germany; 8 Center for Integrative Bioinformatics Vienna, Max F. Perutz Laboratories, University of Vienna, Vienna, Austria; 9 Present Address: Leibniz Institute for Natural Product Research and Infection Biology - Hans Knöll Institute (HKI), Jena, Germany & Center for Sepsis Control and Care (CSCC), Jena, Germany; 10 Present Address: Division of Medical Protein Chemistry, Department of Translational Medicine, Lund University, Malmö, Sweden; 11 Present Address: The Weatherall Institute of Molecular Medicine, University of Oxford, Oxford, UK

## Correction

After publication of the original article [1] the authors noted that the following errors had occurred:

- In the Methods section, it should state that neutrophils were derived from the interphase of 70% and 75% Percoll. In the original article it incorrectly stated 75% and 85% respectively.
  The corrected sentence should read: The cell layer formed at the 70% to 75% interface was collected and washed as before.
- The additional files had been linked incorrectly and the legends did not correspond with the correct documents for Additional files 2, 4, 6, 8 and 10. All the Additional files from the original manuscript have been included with this Correction with the correct files and legends.

The original article has also been updated.

## Additional files

Additional file 1: **Figure S1** Purity analysis of neutrophil isolation. Cellular composition after Percoll gradient purification (A). Neutrophils (CD11b^+^MHCII^−^): 91.8%, monocytes (CD14^+^): 0%, T cells (CD3^+^): 0.7%, DC (CD11c+): 0.1%, B cells (CD19+): 0%, other cells (predominantly FSC^int^ SSC^Hi^ eosinophils): 7.4%. Three multi-color staining panels were used: CD3-FITC, CD19-PE and CD14-APC to distinguish T- and B-cells as well as monocytes; HLA-DP/DQ/DR-FITC and CD11b-Pacific Blue™ for neutrophils and CD11b-Pacific Blue™, CD11c-FITC for DCs. FACS blots of gating strategies used (B). Results from one representative of 5 independent experiments shown. (TIFF 1323 kb)

Additional file 2: **Table ST1** Mapping results. Mapping statistics of neutrophil and *Candida* reads using NextGenMap. (XLS 41 kb)

Additional file 3: **Figure S2** Overview of DEGs over time. The number of DEGs in neutrophils during *C. albicans* infection (A), in PMN-treated *C. albicans* cells (B) and in NET-treated *C. albicans* cells (C) over time. Red indicates up-regulation, blue indicates down-regulation. (TIFF 206 kb)

Additional file 4: **Table ST2 A-C** Differential gene expression analysis of neutrophils. Comprehensive gene expression analysis table of neutrophils infected with yeast (A) and hyphae (B). Each condition was tested against the uninfected PMN control. Union of all 318 protein-coding DEGs in neutrophils (C). (XLSX 19149 kb)

Additional file 5: **Figure S3** Clustering of 318 DEGs in neutrophils infected with *C. albicans*. The entity of protein-coding DEGs in neutrophils affected during a *C. albicans* infection was clustered via QT-Clustering using Mayday based on their fold changes over the time which were z-score normalized for better visualization purposes. The cluster profile hallmarks are indicated. (TIFF 728 kb)

Additional file 6: **Table ST3** Most altered DEGs in neutrophils infected with *C. albicans*. Up- and down-regulated DEGs of neutrophils infected with *C. albicans* yeast and hyphae were sorted by their respective fold change of expression. Positive numbers indicate the ranking amongst up-regulated DEGs; negative numbers indicate the ranking amongst the down-regulated numbers. (PDF 52 kb)

Additional file 7: **Figure S4** Cytokine secretion by neutrophils upon *C. albicans* infection. Neutrophils were analyzed for cytokine release upon 18 h stimulation with thiomersal-killed *C. albicans* hyphae or live *C. albicans* (initially yeast). None of the analyzed cytokines showed a statistically significant difference between stimulation with dead hyphae or live *C. albicans*, indicating that dead hyphae evoke similar responses in neutrophils. Statistical analysis was performed by using a One-way ANOVA with Bonferroni’s post-test (*n* = 5). (TIFF 724 kb)

Additional file 8: **Table ST4 A–C** Differential gene expression analysis of *Candida*. Comprehensive gene expression analysis table of neutrophil-or NET-treated *Candida* cells in yeast form (A) and in hyphae form (B). Respectively to the form, each condition was tested against the unchallenged, but adjusted fungal cell controls. Union of all 797 DEGs in *Candida* cells (C). (XLSX 4327 kb)

Additional file 9: **Figure S5** Overlaps of DEGs in yeast and hypha *C. albicans* challenged with neutrophils. Overlaps of DEGs in *C. albicans* (A) yeast and (B) hyphae challenged with neutrophils throughout the time course. Overlap of morphotype-specific *Candida* response of (C) induced and (D) repressed DEGs. Samples from two independent experiments using different blood donors were analyzed, *n* = 2. (TIFF 353 kb)

Additional file 10: **Table ST5** Differential regulation of arginine metabolism genes in *C. albicans*. DEGs involved in arginine metabolism of yeast and hypha *C. albicans* infecting neutrophils and NETs are displayed. The transcript level is indicated by fold change (log2). (PDF 22 kb)

Additional file 11: **Table ST6 A–D** Quantification results. Comprehensive gene quantification table of neutrophil transcriptome in read counts (A) and in RPKM (B) as well as of *Candida* transcriptome in read counts (C) and in RPKM (D). (XLSX 18989 kb)

Additional file 12: **Figure S6** ROS levels in NET vicinity. ROS produced by in vitro released NETs and neutrophils (PMNs) were quantified by a luminol-based assay over 6 h to test for background ROS due to NET preparation in comparison to stimulated PMNs (A + B). (A): ROS in vicinity of unstimulated, PMA-stimulated, or *Candida*-infected NETs; (B) ROS in vicinity of unstimulated, PMA-stimulated, or *Candida*-infected PMNs. Averages and SD plotted of 3 replicates, *n* = 3. (TIFF 761 kb)
