# Supplementary figures and images for: Dual transcriptome of the immediate neutrophil and Candida albicans interplay
Source: BMC Genomics. 2017 Sep 6;18:696. doi: 10.1186/s12864-017-4097-4 (PMC5585943; doi:10.1186/s12864-017-4097-4)

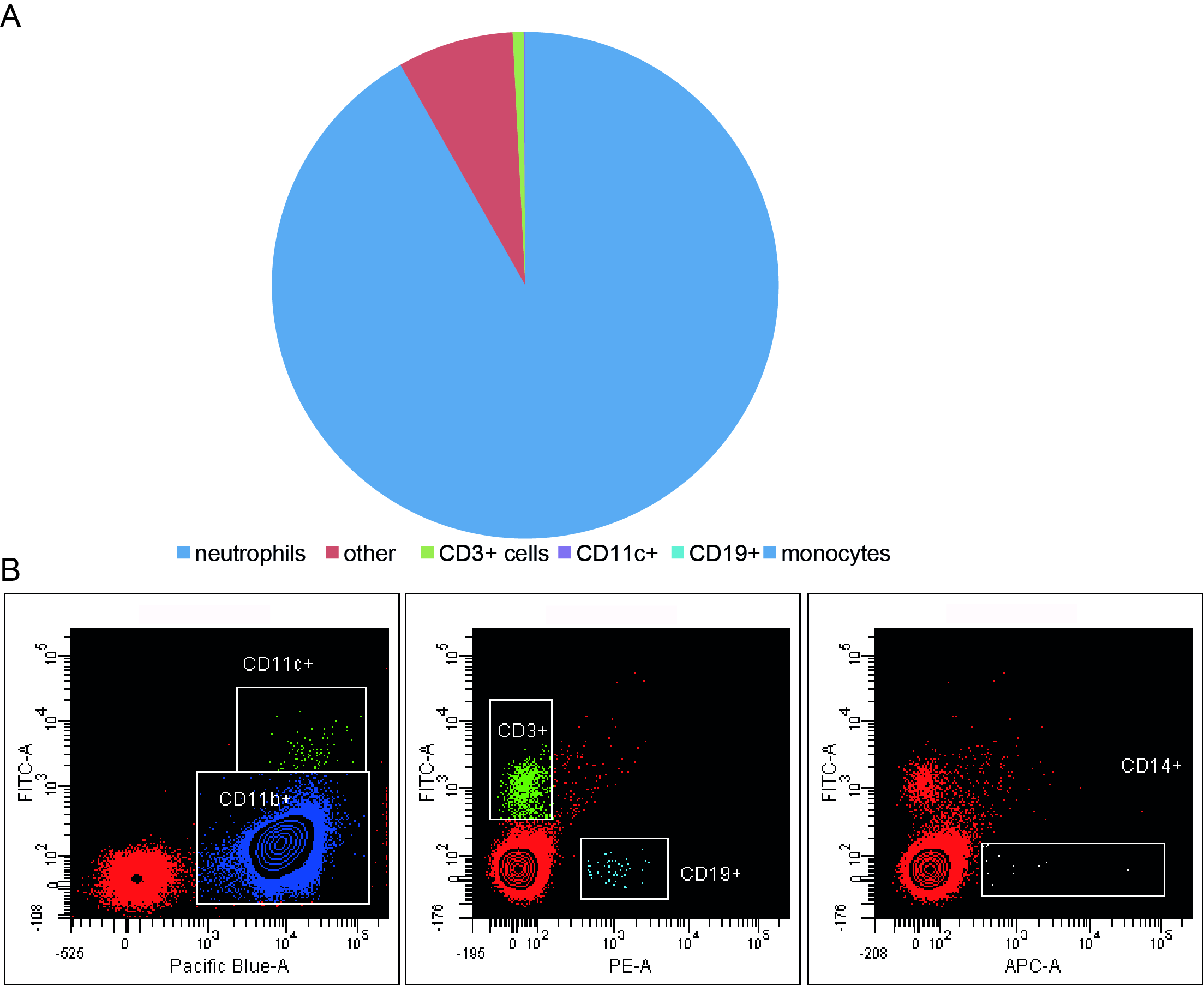

Supplement: Supplementary file 1 — Purity analysis of neutrophil isolation. Cellular composition after Percoll gradient purification (A). Neutrophils (CD11b+MHCII−): 91.8%, monocytes (CD14+): 0%, T cells (CD3+): 0.7%, DC (CD11c+): 0.1%, B cells (CD19+): 0%, other cells (predominantly FSCint SSCHi eosinophils): 7.4%. Three multi-color staining panels were used: CD3-FITC, CD19-PE and CD14-APC to distinguish T- and B-cells as well as monocytes; HLA-DP/DQ/DR-FITC and CD11b-Pacific Blue™ for neutrophils and CD11b-Pacific Blue™, CD11c-FITC for DCs. FACS blots of gating strategies used (B). Results from one representative of 5 independent experiments shown. (TIFF 1320 kb) [file 12864_2017_4097_MOESM1_ESM.tif]

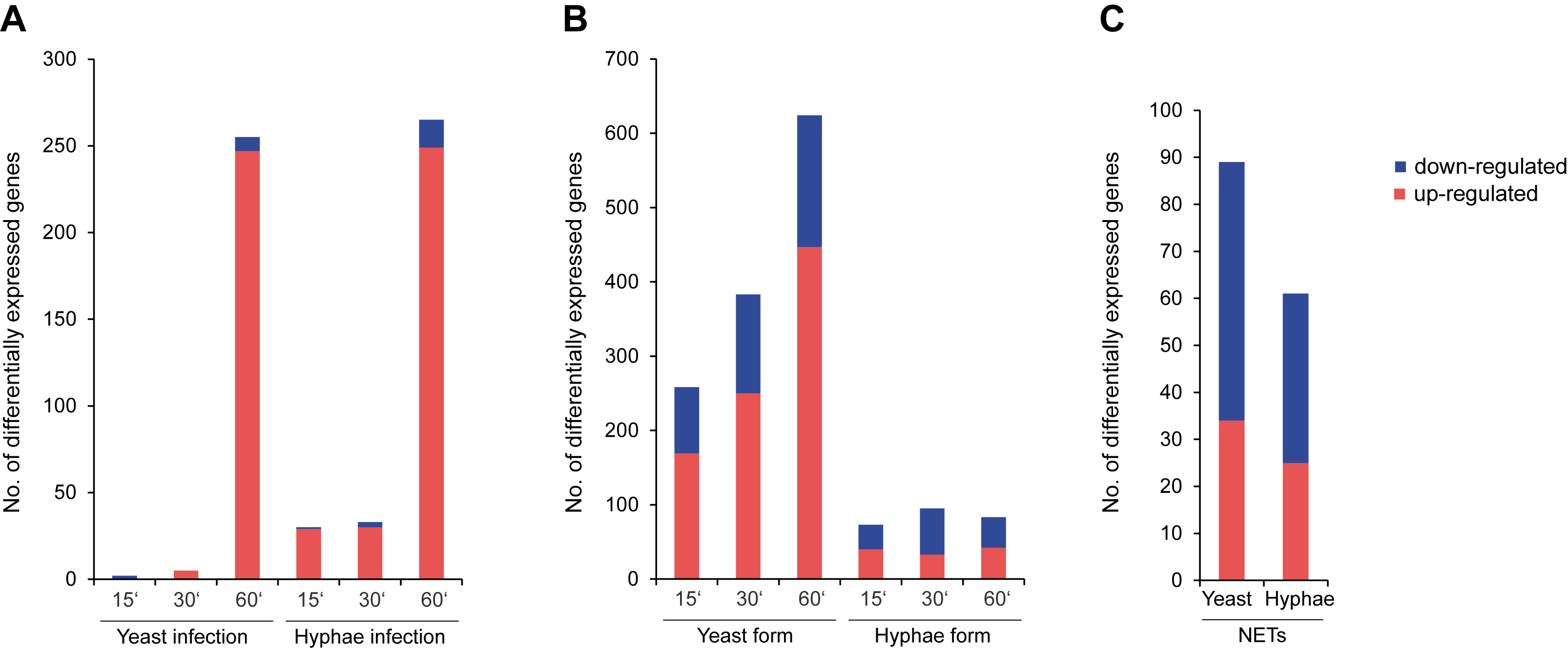

Supplement: Supplementary file 3 — Overview of DEGs over time. The number of DEGs in neutrophils during C. albicans infection (A), in PMN-treated C. albicans cells (B) and in NET-treated C. albicans cells (C) over time. Red indicates up-regulation, blue indicates down-regulation. (TIFF 206 kb) [file 12864_2017_4097_MOESM3_ESM.tif]

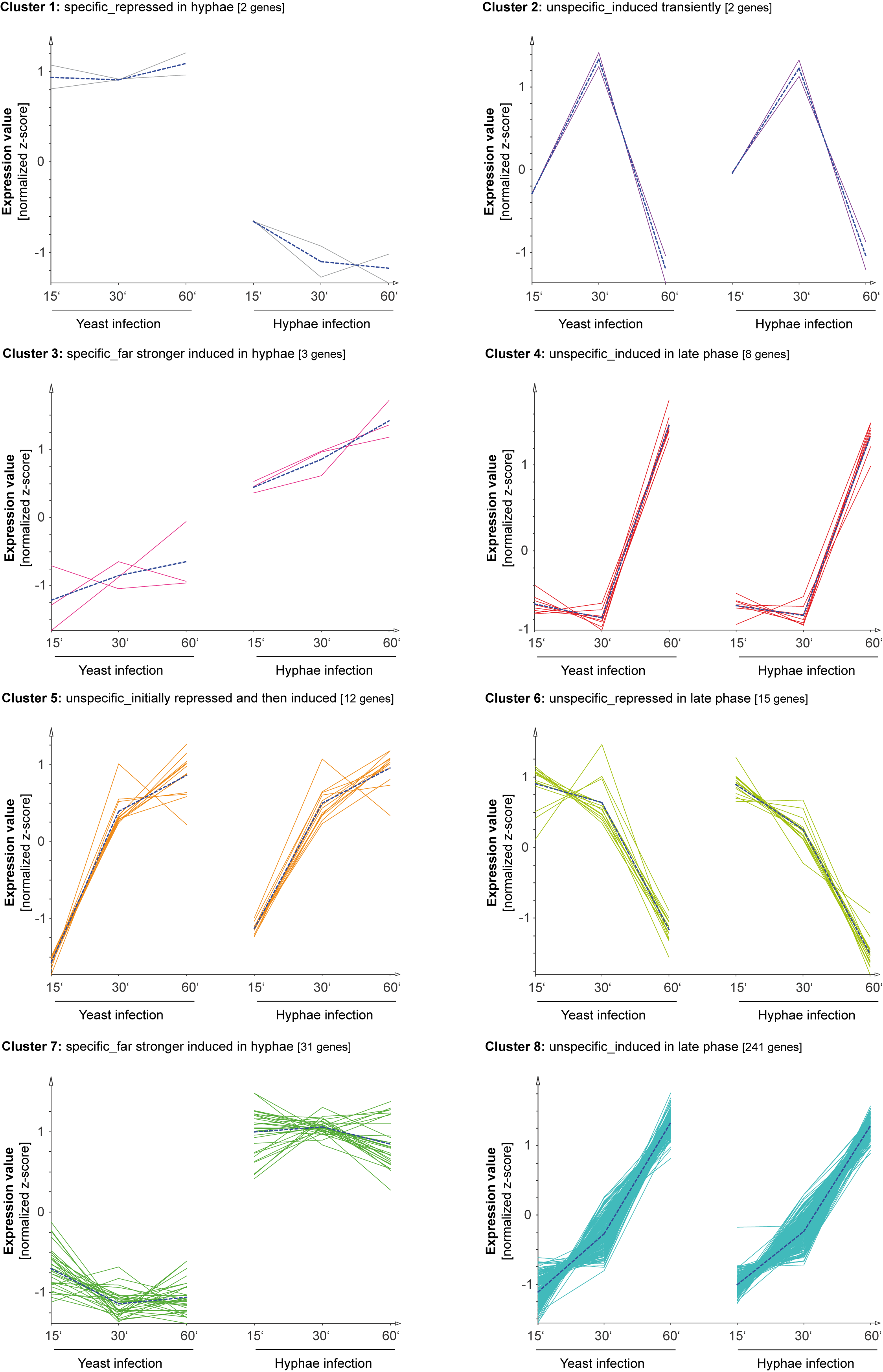

Supplement: Supplementary file 5 — Clustering of 318 DEGs in neutrophils infected with C. albicans. The entity of protein-coding DEGs in neutrophils affected during a C. albicans infection was clustered via QT-Clustering using Mayday based on their fold changes over the time which were z-score normalized for better visualization purposes. The cluster profile hallmarks are indicated. (TIFF 728 kb) [file 12864_2017_4097_MOESM5_ESM.tif]

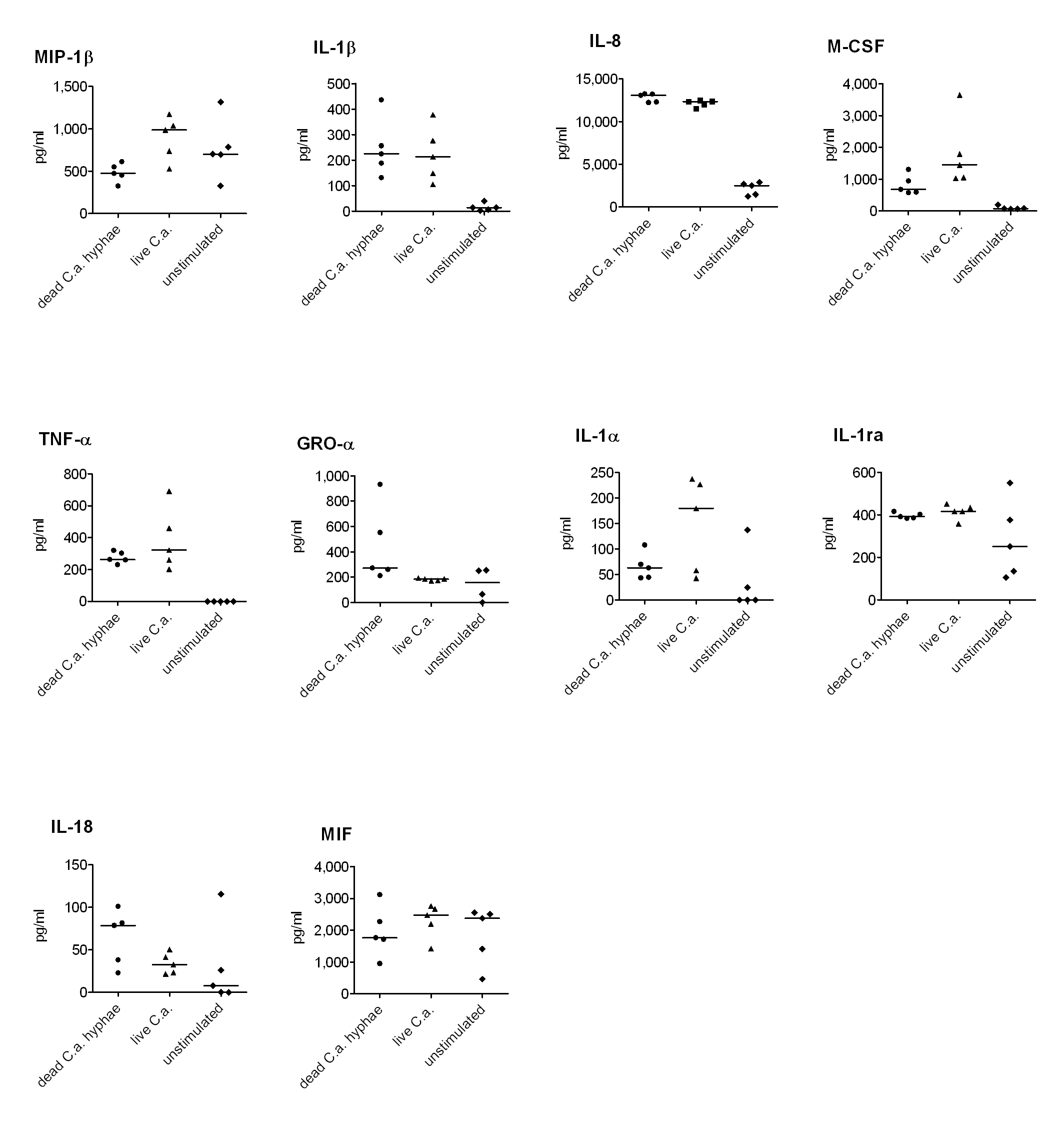

Supplement: Supplementary file 7 — Cytokine secretion by neutrophils upon C. albicans infection. Neutrophils were analyzed for cytokine release upon 18 h stimulation with thiomersal-killed C. albicans hyphae or live C. albicans (initially yeast). None of the analyzed cytokines showed a statistically significant difference between stimulation with dead hyphae or live C. albicans, indicating that dead hyphae evoke similar responses in neutrophils. Statistical analysis was performed by using a One-way ANOVA with Bonferroni’s post-test (n = 5). (TIFF 724 kb) [file 12864_2017_4097_MOESM7_ESM.tif]

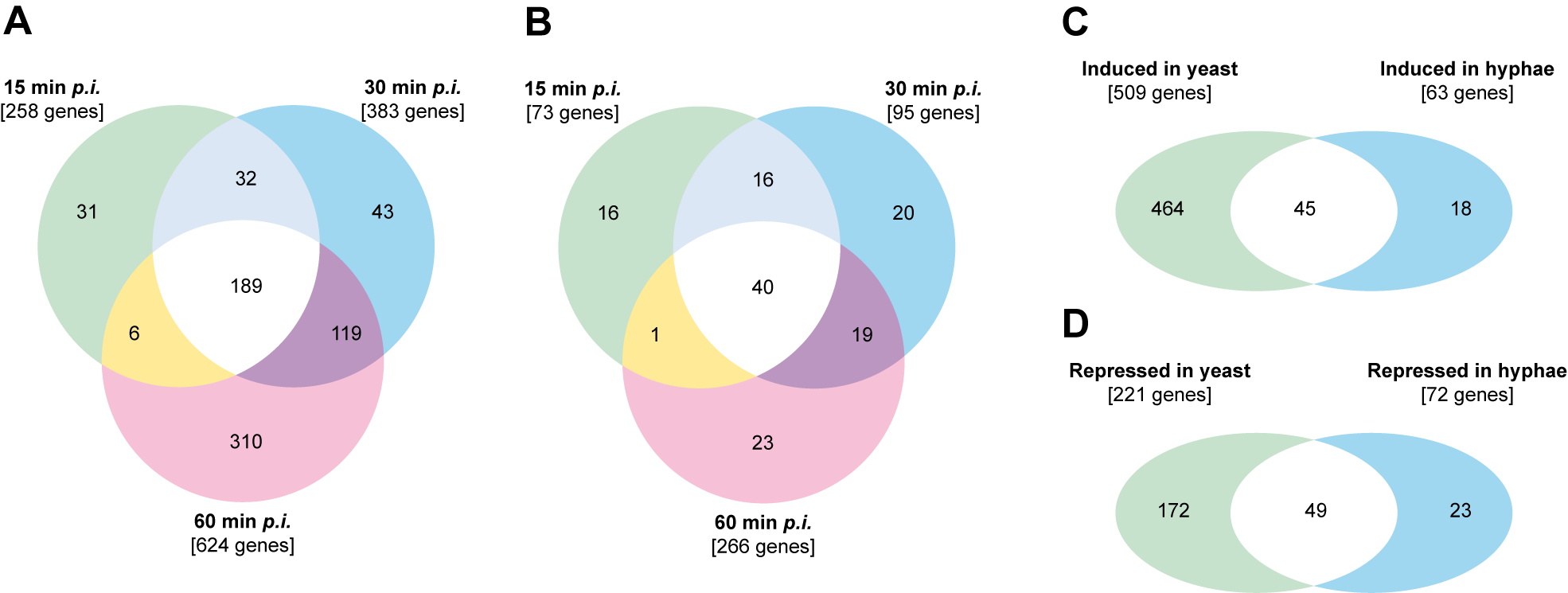

Supplement: Supplementary file 9 — Overlaps of DEGs in yeast and hypha C. albicans challenged with neutrophils. Overlaps of DEGs in C. albicans (A) yeast and (B) hyphae challenged with neutrophils throughout the time course. Overlap of morphotype-specific Candida response of (C) induced and (D) repressed DEGs. Samples from two independent experiments using different blood donors were analyzed, n = 2. (TIFF 353 kb) [file 12864_2017_4097_MOESM9_ESM.tif]

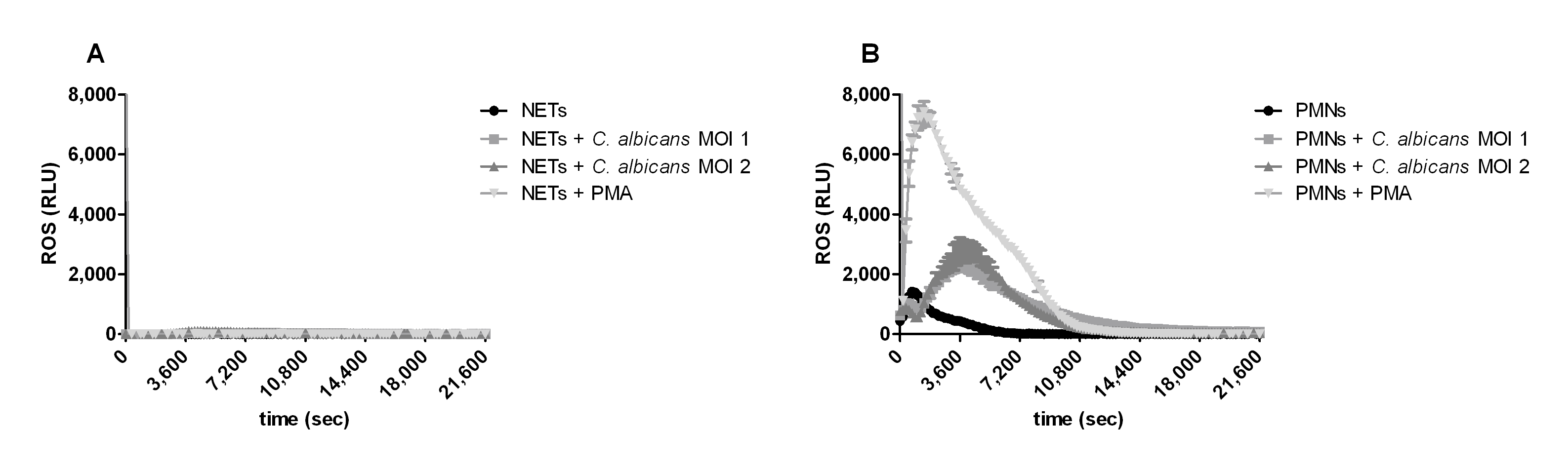

Supplement: Supplementary file 12 — ROS levels in NET vicinity. ROS produced by in vitro released NETs and neutrophils (PMNs) were quantified by a luminol-based assay over 6 h to test for background ROS due to NET preparation in comparison to stimulated PMNs (A + B). (A): ROS in vicinity of unstimulated, PMA-stimulated, or Candida-infected NETs; (B) ROS in vicinity of unstimulated, PMA-stimulated, or Candida-infected PMNs. Averages and SD plotted of 3 replicates, n = 3. (TIFF 761 kb) [file 12864_2017_4097_MOESM12_ESM.tif]
