# Supplementary material for: Dual transcriptome of the immediate neutrophil and Candida albicans interplay
Source: BMC Genomics. 2017 Sep 6;18:696. doi: 10.1186/s12864-017-4097-4 (PMC5585943; doi:10.1186/s12864-017-4097-4)
Supplement: Supplementary file 6 — Most altered DEGs in neutrophils infected with C. albicans. Up- and down-regulated DEGs of neutrophils infected with C. albicans yeast and hyphae were sorted by their respective fold change of expression. Positive numbers indicate the ranking amongst up-regulated DEGs; negative numbers indicate the ranking amongst the down-regulated numbers (PDF 52 kb) [file 12864_2017_4097_MOESM6_ESM.pdf]

Neutrophils infected with	*C. albicans* yeasts			*C. albicans* hyphae		
	Gene	Fold Change [log2]	Rank	Gene	Fold Change [log2]	Rank
15 min	*IL1B*	-2.44	-2	*MYBPC2*	14.99	1
	EGR3	-2.88	-1	*PRTG*	12.45	2
				ANKRD29	10.26	3
				APLN	9.76	4
				HHIP	9.09	5
				ABI2	7.91	6
				RPL3L	7.57	7
				MAP3K9	7.29	8
				NKAPL	7.22	9
				CCDC66	6.54	10
				TUBA1C	-2.63	-1
30 min	*EGR1*		1	*MYBPC2*	14.27	1
	OSM		2	*ALDOB*	13.02	2
	DUSP2		3	*ANKRD29*	10.99	3
	FOS		4	*CCDC169*	10.19	4
	JUNB		5	*APLN*	9.91	5
				HHIP	8.68	6
				MAP3K9	7.88	7
				ABI2	7.86	8
				NKAPL	7.45	9
				CCDC66	6.08	10
				C1orf132	-1.86	-3
				HBD	-2.58	-2
				TUBA1C	-2.95	-1
60 min	*NR4A3*	6.58	1	*ALDOB*	13.53	1
	OLR1	5.45	2	*MYBPC2*	13.49	2
	TRAF1	5.03	3	*ANKRD29*	11.52	3
	CCRL2	4.78	4	*CACNA1H*	10.59	4
	PLAU	4.77	5	*APLN*	9.49	5
	JMY	4.43	6	*MAP3K9*	8.40	6
	C15orf48	4.41	7	*NIM1*	8.30	7
	HS3ST3B1	4.37	8	*NKAPL*	7.05	8
	LIF	4.24	9	*NR4A3*	6.36	9
	IL1A	4.24	10	*STX18*	5.64	10
				NFE2	-1.78	-10
				RAB3D	-1.80	-9
	ITGA5	-1.56	-8	*IRF2BPL*	-1.92	-8
	HSPA6	-1.56	-7	*ITGA5*	-1.97	-7
	DPF2	-1.63	-6	*MYH11*	-2.04	-6
	ZNF217	-1.78	-5	*SETD1B*	-2.06	-5
	CTD-3088G3.8	-1.97	-4	*ZNF217*	-2.10	-4
	NUAK2	-2.07	-3	*NUAK2*	-2.28	-3
	MYH11	-2.24	-2	*HBD*	-2.30	-2
	C1orf132	-2.40	-1	*C1orf132*	-2.39	-1
