# Supplementary material for: Dual transcriptome of the immediate neutrophil and Candida albicans interplay
Source: BMC Genomics. 2017 Sep 6;18:696. doi: 10.1186/s12864-017-4097-4 (PMC5585943; doi:10.1186/s12864-017-4097-4)
Supplement: Supplementary file 10 — Differential regulation of arginine metabolism genes in C. albicans. DEGs involved in arginine metabolism of yeast and hypha C. albicans infecting neutrophils and NETs are displayed. The transcript level is indicated by fold change (log2). (PDF 22 kb) [file 12864_2017_4097_MOESM10_ESM.pdf]

Gene	Fold change in yeasts [log2]				Fold change in hyphae [log2]			
	15 min	30 min	60 min	NETs	15 min	30 min	60 min	NETs
ARG1	1.60	2.84	3.15	0.18	-2.20	-2.24	-0.61	2.33
ARG3	1.67	2.57	3.09	0.20	-2.51	-1.55	-0.18	2.31
CAR1	-0.15	-0.48	-1.72	0.32	2.98	2.69	2.26	-0.52
CAR2	-0.47	-1.74	-2.38	-0.08	2.11	2.21	1.46	-0.82
	Neutrophils				Neutrophils			
